# Functional Thermoplastic Materials from Derivatives of Cellulose and Related Structural Polysaccharides

**DOI:** 10.3390/molecules20045487

**Published:** 2015-03-27

**Authors:** Yoshikuni Teramoto

**Affiliations:** Course of Applied Life Science, Faculty of Applied Biological Sciences, Gifu University, 1-1 Yanagido, Gifu 501-1193, Japan; E-Mail: teramoto@gifu-u.ac.jp

**Keywords:** cellulose, polysaccharides, derivatization, material functionality, thermal transition, polymer blend, graft copolymerization

## Abstract

This review surveys advances in the development of various material functionalities based on thermoplastic cellulose and related structural polysaccharide derivatives. First, the dependence of thermal (phase) transition behavior on the molecular composition of simple derivatives is rationalized. Next, approaches enabling effective thermoplasticization and further incorporation of material functionalities into structural polysaccharides are discussed. These approaches include: (a) single-substituent derivatization, (b) derivatization with multi-substituents, (c) blending of simple derivatives with synthetic polymers, and (d) graft copolymerization. Some examples addressing the control of supramolecular structures and the regulation of molecular and segmental orientations for functional materials fabrication, which have especially progressed over the past decade, are also addressed. Attractive material functions include improved mechanical performance, controlled biodegradability, cytocompatiblity, and optical functions.

## 1. Introduction

**Scheme 1:**
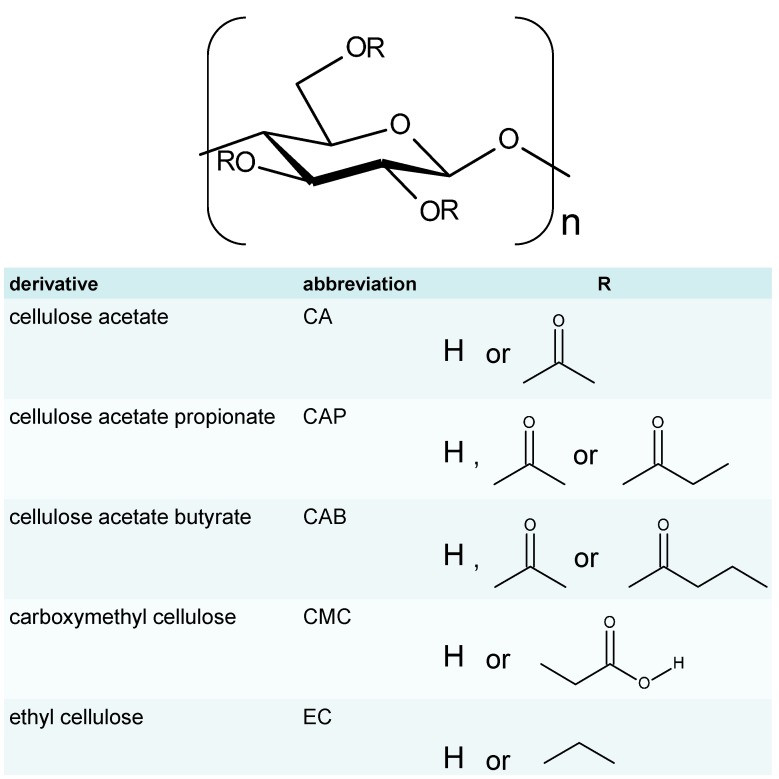
Representative commercially available cellulose derivatives.

Cellulose derivatives are a class of compounds that originated with nitrate (celluloid), the first man-made thermoplastic to be industrialized in the 19th century, and still retain their industrial importance because of their spinnability, film formability and transparency, strength and tenacity, sorption performance, and other useful properties. Representative commercially available cellulose derivatives and their formulae are listed in Scheme 1. Currently, the conversion of cellulose to advanced materials has attracted considerable attention in connection with green and sustainable industrial development [1,2,3,4,5].

In general, strong intermolecular and intramolecular hydrogen bonding in unmodified cellulose and other structural polysaccharide molecules gives rise to a theoretical glass transition temperature (*T*_g_) exceeding their thermal decomposition point, limiting thermal processability. Derivatization enables the modification of original bulk material properties such as thermal behavior through relatively simple reactions that effectively exploit side-group reactivity. However, the relationship between molecular structure and physical properties and the principles governing the derivatization need to be elucidated in detail.

Numerous investigations have addressed cellulose chemical modifications such as ester synthesis [1,6], graft copolymerization [7,8], and nanocellulose formation [9]. While intentionally avoiding studies on the synthetic aspects of cellulose and related structural polysaccharide derivatives, the present review covers the development of material functionalities for systems containing thermoplastic derivatives of cellulose and related polysaccharides because few reviews have dealt with this subject. Therefore, a systematic interpretation of fundamental thermal properties of simple derivatives is discussed first (Section 2.1), and various material functionalization approaches developed over the last decade to derivatize cellulose and related polysaccharides as thermoplastic substances are subsequently reviewed. For convenience, these modifications, which facilitate thermoplasticization and further material functionalization of structural polysaccharides, are grouped into four classes: (a) single-substituent derivatization (Section 2), (b) derivatization with multiple substituents (Section 3), (c) blending of simple derivatives with synthetic polymers (Section 4), and (d) graft copolymerization (Section 5). Highlighted material functionalities consist of improved mechanical performance, controlled biodegradability, cytocompatiblity, and optical functions. Specific progress in the fabrication of functionalized bulk materials has also rapidly occurred over the past decade as a result of the control of supramolecular structures and the regulation of molecular and segmental orientations. This topic will be reviewed separately in Section 6.

## 2. Single Substituent Derivatives

### 2.1. Fundamental Aspects of Thermal Properties and Phase Behavior

For polymer solutions, intrinsic viscosity [η] and molecular weight *M* are related by the Mark-Houwink-Sakurada equation as follows:

[η] = *K*_m_*M^a^* (1)

where *K*_m_ and *a* are parameters characteristic of the polymer/solvent combination at a given temperature. Cellulose derivatives generally show exponent *a* values ranging from 0.8 to 1.0, indicative of semi-rigid polymer chains. Their properties are dominated by a balance between the semi-rigidity of the cellulosic trunk and the nature of side chains. Thermal properties of polymer materials remarkably depend on the backbone flexibility, steric hindrance, and chain-chain polar interaction. The semi-rigid cellulosic main chain is essentially internally plasticized by the introduction of bulky side-chain groups leading to intermolecular hydrogen bond scission.

**Figure 1:**
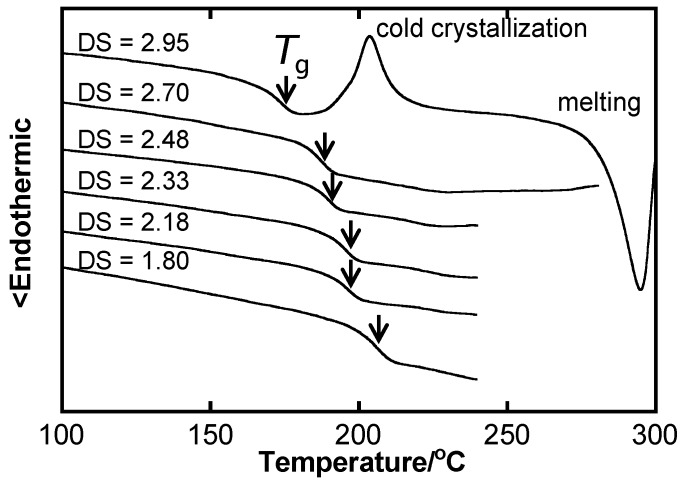
DSC thermograms of CA displaying different DS values obtained during the second heating scan. Arrows indicate *T*_g_ positions taken as midpoints of heat flow discontinuity.

In general, for cellulose derivatives, thermal and dissolution properties are determined by the degree of substitution (DS) defined as the average number (up to three) of substituted hydroxyl groups per anhydroglucose unit. As demonstrated by differential scanning calorimetry (DSC) thermograms (Figure 1), *T*_g_ decreases with increasing DS for cellulose acetate (CA). However, triacetylated cellulose (DS = 2.95) tends to crystallize easily because of its highly symmetric structure. Irrespective of side-chain moieties, cellulose derivatives presenting a low DS (~1.0) often show water solubility, and solubility in hydrophobic organic solvents generally increases with increasing DS.

Cellulose derivatives presenting a DS value of approximately 3 exhibit a complicated thermal transition behavior depending on the side chain size. Thermal (melt) processability is achievable by incorporating bulky ester substituents into the cellulose backbone [1,10,11,12,13,14]. “Melt processing,” however, implies the application of softening behavior including phenomena of glass transition, liquid crystalline transition, and crystallite melting, if any. It is therefore safe to put “melt” into “thermal” processing in order to avoid confusion with melting of crystallites in a thermodynamic sense.

**Figure 2:**
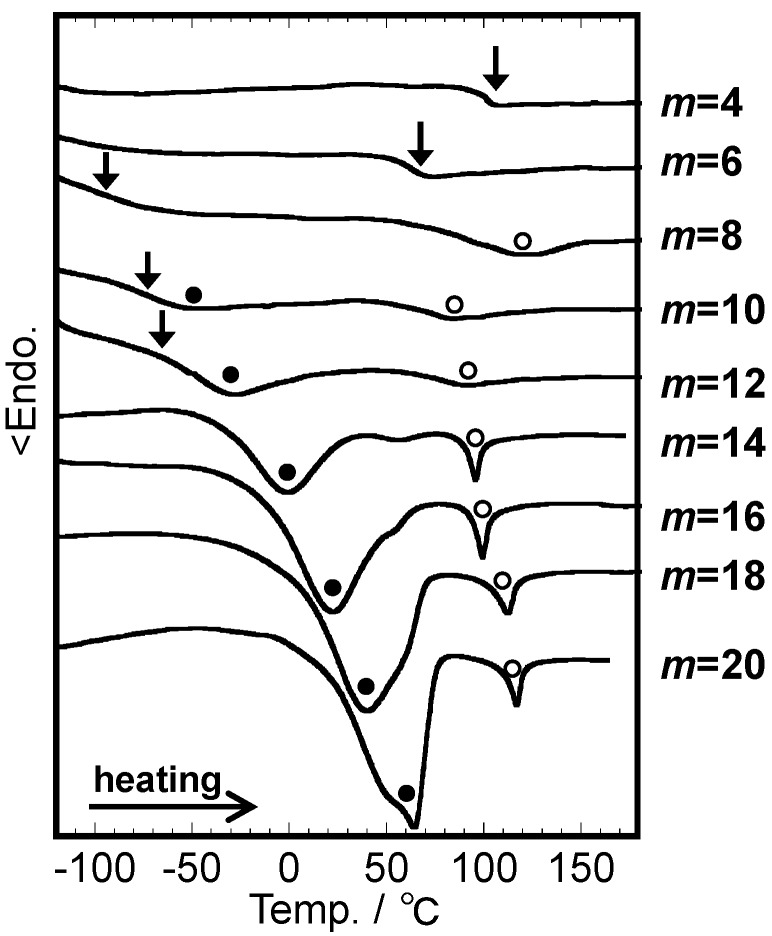
DSC thermograms of cellulose triesters presenting different *m* values obtained during the second heating scan. Arrows mark *T*_g_ positions while the filled and open circles represent *T*_m_ and *T*_i_ values, respectively.

The thermally processable temperature of cellulose triesters decreases rapidly when the ester substituent becomes bulkier. Figure 2 shows typical DSC thermograms acquired during the second heating scan at 20 °C/min of cellulose triesters bearing linear side chains as a function of side chain length *m*, where *m* is defined as the number of the carbon atoms forming the side chain skeleton, just after quick quenching (cooling at ~80 °C/min) from the isotropic state (200 °C). Interpreting the thermal events observed by DSC analysis often causes confusion. The dramatic lowering of baseline shift temperatures may be considered as a decrease in *T*_g_ for substituents ranging between acetate (*m* = 2) and hexanoate (*m* = 6). *T*_g_ remains more or less constant (or even rises slightly) for substituents displaying longer chains. The drop in *T*_g_ may be modeled as an internal plasticization process of all cellulosic molecules containing methylene groups, almost conforming with the Fox relationship [15]. The failure of waxy triesters (*m* ≥ 10, Figure 2) to follow this rule may originate from the formation of highly-ordered domains (pseudo-crystallites) mainly comprising longer side chains and the development of a liquid crystalline (LC) phase [15,16]. Eventually, the glass transition appears more latent, and (side-chain pseudo-) crystallite melting (*T*_m_) and molecular thermotropic LC isotropization (*T*_i_) become more easily detectable (Figure 2). The LC phase of the cellulose esters, characterized by a low birefringence and high viscosity compared to cholesteric phases, is classified as a columnar structure, in which molecules adopt a two-dimensional hexagonal packing order in the plane normal to the chain axis with two chains being included in a unit lattice [16].

**Figure 3:**
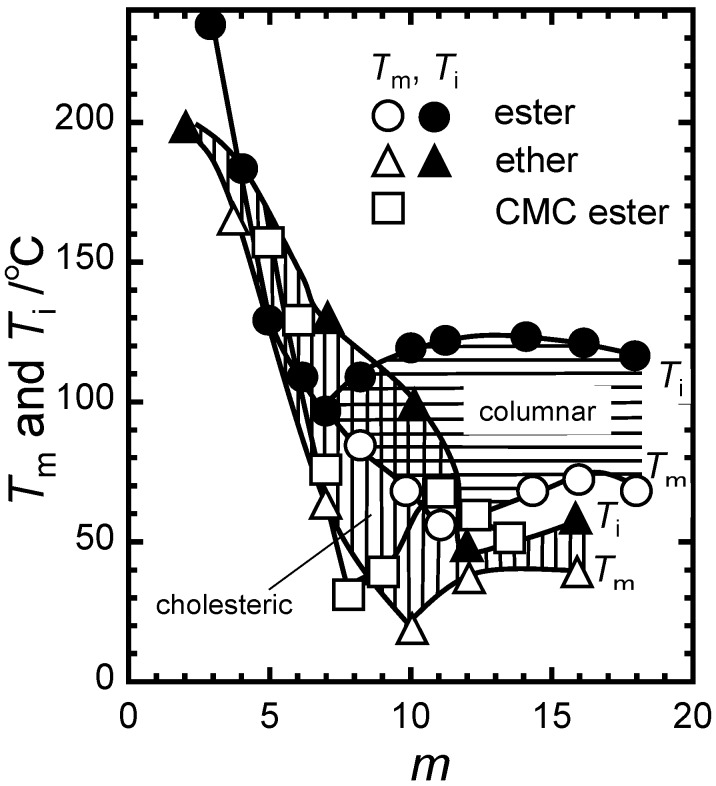
Melting temperature *T*_m_ and isotropization temperature *T*_i_ of normal alkyl ester and ether derivatives of cellulose. Ester derivatives of tri-*O*-carboxymethyl cellulose (CMC) hardly form a mesophase regardless of *m*. Reproduced with permission from ref. [16]. Copyright Takeshi Fukuda, Kyoto University (1991).

Fukuda *et al.* have elegantly summarized the complicated thermal transition behavior of tri-substituted cellulose derivatives in terms of thermotropic LC formation [16]. Figure 3 shows the thermal transition temperatures of cellulose triesters as well as cellulose triethers and tri-*O*-carboxymethyl cellulose (CMC) triesters as a function of *m*. For example, *m* = 7 represents heptyl cellulose (Cell-O-C_7_H_15_), cellulose heptanoate (Cell-O-CO-C_6_H_13_), and carboxymethyl cellulose pentyl ester (Cell-O-CH_2_-COOC_5_H_11_). In all cases, the melting temperature *T*_m_ decreases similarly with increasing *m*. Alkyl ethers organize into a cholesteric phase (vertically hatched region) while alkyl esters give a columnar phase (horizontally hatched region). However, CMC derivatives hardly exhibit any liquid crystallinity regardless of *m*. According to the Flory theory, cholesteric phases are believed to originate essentially from “hard interactions,” which represent orientation-dependent excluded-volume effects between “hard” rods or Kuhn segments exclusively interacting through short-range repulsions that preclude the intrusion of one rod or segment into the space occupied by another [17]. In the columnar phase, it is important to account for energetic contributions of “soft interactions,” that is the effects of intermolecular attractive forces, such as van der Waals forces between alkyl side chains, similar to the ones between solvent-solute pairs, in addition to hard interactions. In this regard, CMC derivatives may be considered to miss these mesophase-stabilizing hard and soft interactions.

### 2.2. Systematic Studies of Related Polysaccharides

Associated with the extensive studies of cellulose derivatives described above, derivatives of other carbohydrates have prompted similar interest for the formulation of thermal properties. Provided in abundance by marine crustaceans, chitin is a representative animal-derived structural polysaccharide. Structurally similar to cellulose, it may be regarded as a cellulose molecule in which the hydroxyl group at the C2 position is replaced by an acetylamino group. Chitosan is a collective name for chitin deacetylated to different degrees. Fully deacetylated chitin is soluble in dilute aqueous acid solutions, whereas its ~50% deacetylated counterpart readily dissolves in neutral water. Chitinous compounds differ from cellulosics by the presence of (acetyl) amino groups in their structure, resulting in distinct biological functions, such as bio-assimilability and antibacterial activity. Chitin and chitosan are therefore expected to find wide application in medical, pharmaceutical, food, and textile industries.

**Figure 4:**
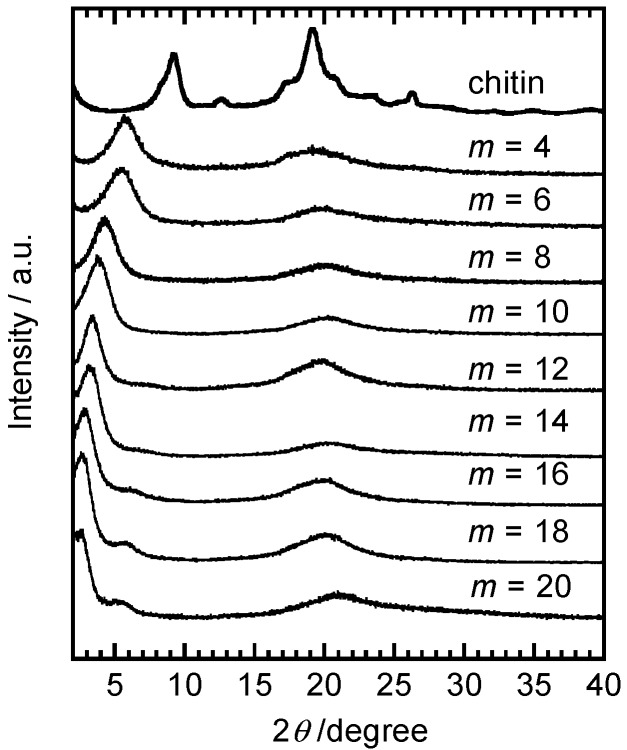
WAXD profiles of chitin and its diester derivatives (*m* = 4–20) at 20 °C. Reprinted with permission from ref. [18]. Copyright 2006 American Chemical Society.

Teramoto *et al.* synthesized a series of single-substituent chitin diester derivatives bearing normal acyl groups (Chitin-O-CO-C*_m_*_−1_H_2 m−1_) using pyridine, *p*-toluenesulfonyl chloride, and normal alkanoic acid in a *N*,*N*-dimethylacetamide (DMAc)–LiCl homogeneous system [18]. The products (DS = 1.7–1.9) showed an *m*-dependent thermal transition behavior. Specifically, no evident transition was observed for *m* = 4–10. On the other hand, a glass transition was detected for *m* = 12 and 14, and a pseudo-first-order phase transition was recorded for *m* = 16–20. These transitions usually occurred below room temperature by DSC. Wide-angle X-ray diffractometry (WAXD) patterns at 20 °C displayed a sharp diffraction peak (2θ = 2°–7°) and diffuse halo (2θ ≈ 20°) for the chitin diesters (Figure 4). The *d*-spacing (1.5–3.6 nm) increased with increasing *m* to yield two stages of mutually different increasing rates, consistent with the systematic *m*-dependence of the period of the main chain layered structure. The molecular assembly of the chitin diesters exhibited “dual mesomorphy”: nematic ordering for the semi-rigid carbohydrate trunk and smectic ordering for the flexible side chains. In relation to this depiction, the typical phase diagram of the already-described cellulose ester consisted of crystalline solid, mesomorphic, and isotropic regions. In contrast, chitin diester bulk samples were continually birefringent at any temperature during heating and eventually underwent thermal degradation without exhibiting any evident flow. In addition, chitin diesters showed poor solubility in common organic solvents, such as acetone, tetrahydroflan, dimethyl sulfoxide, DMAc, toluene, and chloroform. These properties of the chitin diesters are in stark contrast to those of the cellulose ester series [12,15,16] and aromatic liquid-crystalline polyesters [19], in which the acylation was conducted primarily to plasticize the respective rigid main-chain polymers. Therefore, the acylation hardly produced an “internal plasticization” of the rigid chitinous polymer. A quasi-complete diesterification may induce a suitable conformational symmetry around the chitin backbone axis and growth in the lateral dimension via amide groups, which are prone to form a strong intermolecular hydrogen bonding, thereby resulting in the nematic alignment of the main chains as rather stiffer trunks through side chain interlocking.

**Figure 5:**
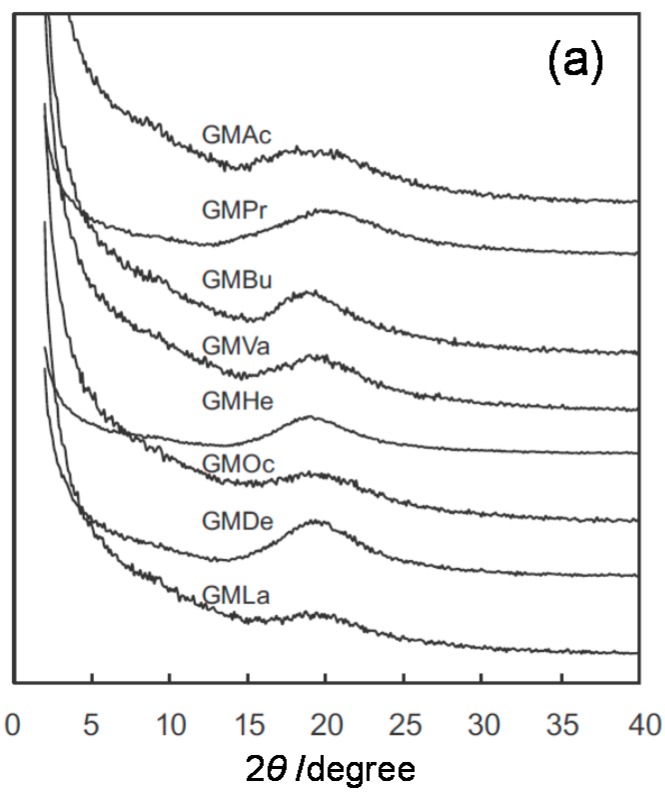

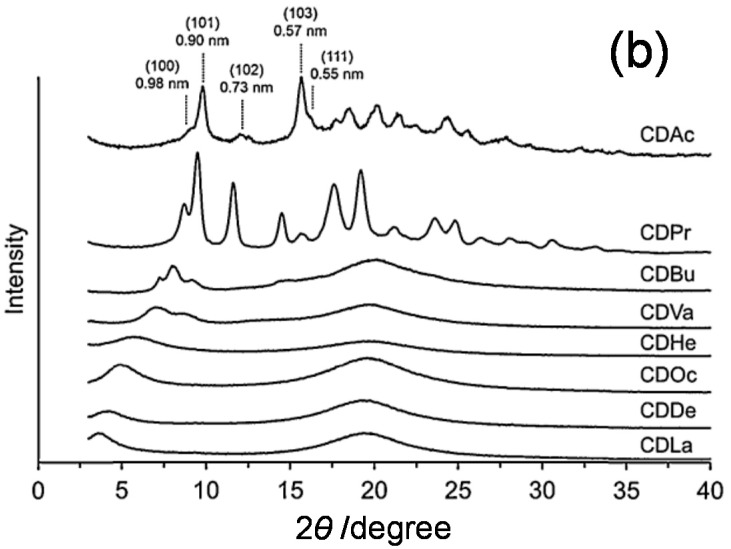
WAXD profiles of (**a**) konjac glucomannan (GM) and (**b**) curdlan (CD) ester derivatives. Abbreviations: Ac, acetate (*m* = 2); Pr, propionate (*m* = 3); Bu, butyrate (*m* = 4); Va, valerate (*m* = 5); He, hexanoate (*m* = 6); Oc, octanoate (*m* = 8); De, decanoate (*m* = 10); La, laurate (*m* = 12). Reproduced with permission from refs. [21] and [22] for (a) and (b), respectively. Copyright 2013 and 2014 Elsevier Ltd.

Defined as heteroglycans consisting of xylose, mannose, arabinose, glucose, galactose, and 4-*O*-methyl-d-glucuronic acid, hemicelluloses constitute about 25%–35% of plants. They exhibit high structural diversity by incorporating different types of side chains and glycosidic linkages in their backbone. Together with cellulose, they form a complex structure in cell walls, providing structure and rigidity to plants. A predominant type of hemicellulose called xylan mainly consists of β-1,4-linked d-xylopyranose units and displays various chemical structures, which may even be branched, depending on its source. Iwata *et al.* have synthesized a series of high-molecular weight xylan esters comprising different alkyl side chain length (*m* = 2–12) by homogeneous reactions [20]. The esterification improved the solubility of xylan in chloroform, but thermal transitions, such as melting and glass transitions, were not detected by DSC between −20 °C and 250 °C. WAXD profiles showed two main diffraction peaks, similarly to those of the chitin diesters (Figure 4). Mechanical properties of the xylan esters were dependent on the alkyl chain length. Specifically, the tensile strength of solution-cast films decreased with increasing *m*, and xylan butyrate (*m* = 4) exhibited the highest tensile strength (29 MPa). On the other hand, the elongation at break increased with increasing *m* and amounted to 19%, 46%, and 44% for xylan butyrate (*m* = 4), decanoate (*m* = 10), and laurate (*m* = 12), respectively. Subsequently, a similar derivatization was applied to konjac glucomannan (GM), a hemicellulose comprising β-1,4-linked d-glucose and d-mannose residues in its main chain with branching through the C3 carbon of glucosyl or mannosyl residues [21], and curdlan (CD), a linear β-1,3-d-glucan [22]. Similarly to the xylan esters, the tensile strength and Young’s modulus tended to decrease while the elongation at break increased with increasing *m* for both esterified hemicellulose series. Thermally pressed GM propionate (*m* = 3) and butyrate films (*m* = 4) presented the highest tensile strength (*ca*. ~53 MPa) while the GM laurate film (*m* = 12) showed the highest elongation at break (426%). As displayed in Figure 5, WAXD profiles revealed that low symmetry and branched GM derivatives exhibited completely amorphous structures. In contrast, CD derivatives bearing shorter acyl side chains (*m* ≤ 6) rapidly crystallized, in agreement with DSC measurements. No LC formation was observed for these hemicellulosic systems.

## 3. Derivatization with Multiple Substituents

Ductility and toughness are generally incompatible in cellulose-based materials. Single bulky substituents not only promote the expression of thermoplasticity but also tend to soften cellulose derivatives [23], as may be deduced from their thermal behavior and the mechanical data of hemicellulose derivatives (see above, Section 2.2). This suggests that size and DS control of single substituents are not sufficient to balance the mechanical performance (strength) and thermal moldability of cellulose derivatives. The thermotropic nature may also lead to difficulties in their application as structural bulk materials. Although mixed cellulose acetate propionates (CAPs) and butyrates (CABs) have been synthesized commercially (Scheme 1), these materials are virtually unprocessable by thermal methods without the addition of large amounts of external low-molecular-weight plasticizers. However, the introduction of small amounts of ester substituents larger than C4 into CA results in a rapid decline in the thermally processable temperature [24]. This incorporation of multiple substituents has been widely conducted to improve both the mechanical performance and thermal processability and to add specific material functionalities.

**Figure 6:**
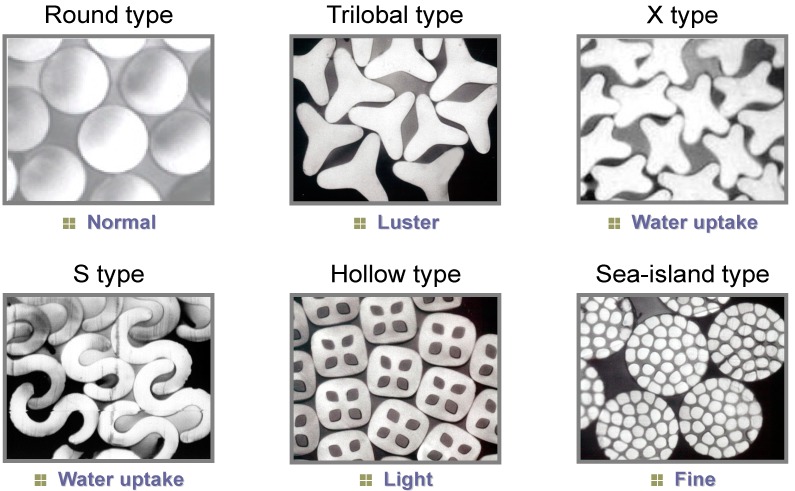
Various fiber cross-sections obtained for the first melt-spun cellulose fiber “Foresse^®^.” Reproduced with permission from ref. [25]. Copyright 2014 Toray Industries, Inc.

**Figure 7:**
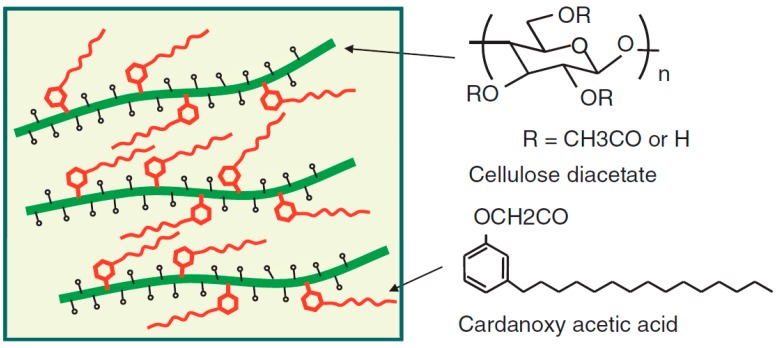
Hypothetical structure of cardanol-bonded CDA. Reproduced with permission from ref. [26]. Copyright 2011 The Society of Polymer Science, Japan (SPSJ).

Industries have recently deployed tremendous efforts to develop new cellulose-based thermoplastic polymers combining excellent thermal processability and tunable mechanical performance by multi-substitution. Aranishi *et al.* (Toray Industries, Inc., Tokyo, Japan) developed the world’s first melt-spun cellulosic fiber Foresse^®^ [25] while the production of conventional cellulosic filaments, such as viscose rayon and cellulose acetate, requires harmful organic reagents and solvents. Substituent and DS optimization gave rise to cellulose mixed esters displaying excellent thermal flowability and fiber mechanical properties. This melt spinning process readily leads to a wide variety of fiber cross sections, such as trilobal, hollow, and sea-islands-type conjugated fibers (Figure 6). The fabric obtained from the cellulosic fiber exhibits soft touch, hygroscopicity, good color, and luster, making it suitable for textiles, such as apparel. Iji *et al.* (NEC Corporation, Tokyo, Japan) produced cellulose-based bioplastics by derivatization of cellulose diacetate (CDA) using 3-pentadecylphenoxy acetic acid (PAA) chloride (Figure 7) called a modified cardanol [26]. Derived from cashew nut shells, cardanol is a phenol derivative bearing a linear unsaturated hydrocarbon side chain (carbon number: 15) [26]. Esterification of CDA with PAA chloride produced thermoplastic PAA-bonded CDA exhibiting high tenacity (elongation at break exceeding 10% while keeping maximum bending strength (>80 MPa)), heat resistance, and water resistance.

**Figure 8:**
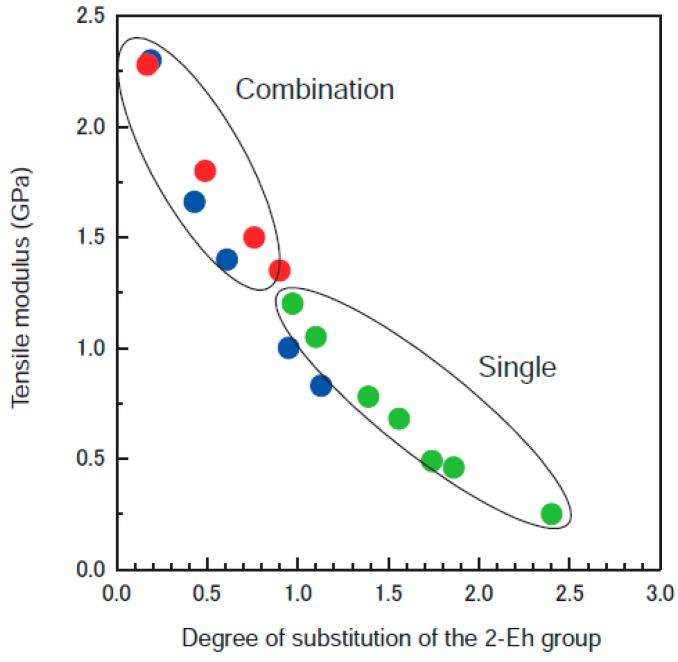
Tensile strength as a function of substitution using methyl/2-ethylhexanoyl (2-Eh) (blue), acetyl/2-Eh (red), and 2-Eh groups (green). Reprinted with permission from ref. [23]. Copyright 2012 FUJIFILM Corporation.

Sawai *et al.* (FUJIFILM Corporation, Tokyo, Japan) precisely demonstrated that the size and number of introduced multi-substituents, such as combinations of methyl/2-ethylhexanoyl (2-Eh) and acetyl/2-Eh groups, significantly enhanced cellulose derivative plasticity [23]. An association of small and large substituents also dramatically improved the balance between mechanical properties and plasticity (Figure 8). Furthermore, the addition of ether/ester groups together boosted the impact strength of the derivatives, in good correlation with the degree of prevalence of soft segments estimated by dynamic mechanical analysis (DMA) and solid-state NMR. These results may stem from three factors related to the volume and motion of the cellulosic molecular chains. (i) A constant number of molecular motion elements can absorb energy in high-speed destruction tests, such as impact tests at room temperature. (ii) The increase in the degree of freedom in space promotes molecular motions within gaps in response to impact. (iii) The weakened hydrogen bonds led to a symmetrical uniaxial cylindrical structure comprising cellulosic molecules. The materials were processed by injection molding and showed good rheological and processing characteristics. Building on these results, Yao *et al.* (Fuji Xerox Co., Ltd., Tokyo, Japan) proposed multisubstituted cellulose derivatives bearing some residual hydroxyl groups to develop highly thermally processable materials without reduction in mechanical strength and heat resistance [27]. An exploration of appropriate halogen-free flame retardants as additives highlighted compatible composite systems combining the derivatives with acrylonitrile butadiene styrene (ABS) resin at adequate molar ratio, molecular weight, and melt viscosity. The composites were eventually mounted as components for business machines. Similarly, a good thermoplasticity–modulus balance was obtained by mixed esterification for paramylon, a storage β-1,3-glucan polysaccharide for *Euglena gracilis* [28].

**Figure 9:**
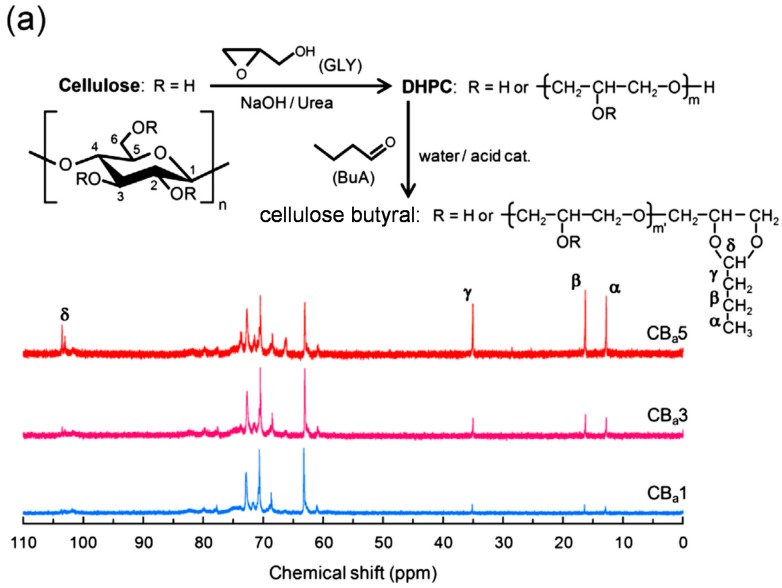

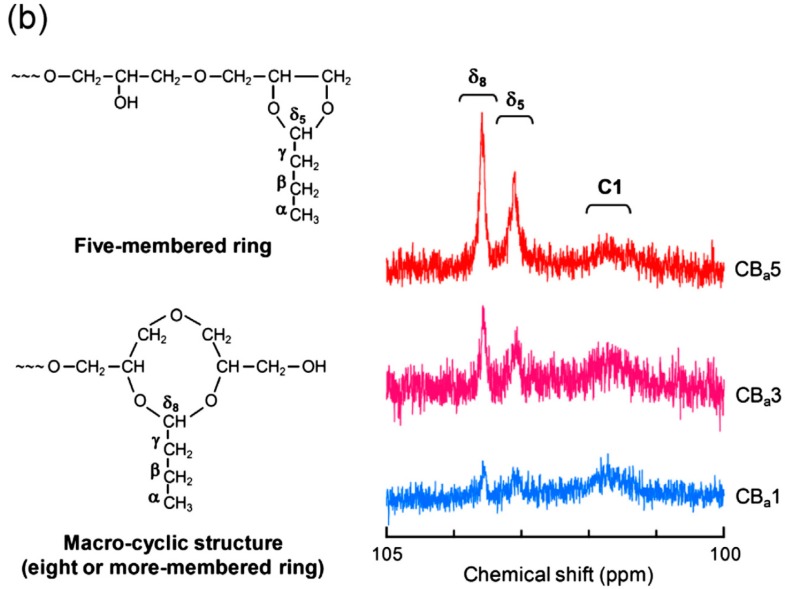
(**a**) Derivatization reactions and ^13^C-NMR spectra of cellulose butyral products in pyridine-*d*_5_ showing the butyral carbon assignment; (**b**) enlarged ^13^C-NMR spectra between 100 and 105 ppm and peak assignments. The DHPC precursor comprised 81.1 wt % dihydroxypropyl unit. Butyral molar substitutions of the products are 0.20, 0.94, and 3.60 for CB_a_1, CB_a_3, and CB_a_5, respectively. Reprinted with permission from ref. [29]. Copyright 2014 Elsevier Ltd.

Furthermore, practical and natural polymer materials have inspired researchers to develop derivatives displaying interesting functions as bulk materials. Chang *et al.* fabricated high performance films using cellulose butyral synthesized from native cellulose [29,30] based on the commercial production of poly(vinyl butyral) (PVB) from poly(vinyl alcohol) [31]. Bulk PVB acts as an interlayer in laminated glass because the film adheres well to glass surfaces and provides excellent mechanical resistance to the laminate against breakage [32]. Acetalization generates 1,3-dioxane rings, which may contribute to the impact resistance and toughness of PVB films. Inspired by the PVA-based commodity, the researchers attempted to create these interlayers from cellulose. As summarized in Figure 9, a two-step synthesis was adopted to produce the cellulose butyral derivative. Specifically, etherification of cellulose with glycidol in NaOH/urea aqueous solution [33] gave *O*-(2,3-dihydroxypropyl) cellulose (DHPC), which underwent butyralization to generate the desired derivative [29]. The product displayed at least two annular structures, such as five- and eight-membered rings, on the side chains and was easily molded into thin films by hot pressing. In addition to being amorphous (*T*_g_ < 25 °C) and highly transparent, the cellulose butyral films conveniently provided a slightly lower refractive index than conventional glass (~1.51). Despite their insolubility in water, they showed a good adhesive property to glass plates because of their amphiphilic character. DMA results revealed that the original semi-rigid cellulosic molecule was highly plasticized by a surrounding layer of dihydroxypropyl sequences. The flexible polyether side-chains were primarily responsible for the ductile behavior observed in DHPC and cellulose butyral films in tensile tests. The butyral group improved the toughness of the DHPC films during stretching because chain entanglement was more effective in the ring structures. These cellulose butyral films presented comparable optical and mechanical performances to commercial PVB films, suggesting their potential applicability as interlayers for laminated glass.

**Figure 10:**
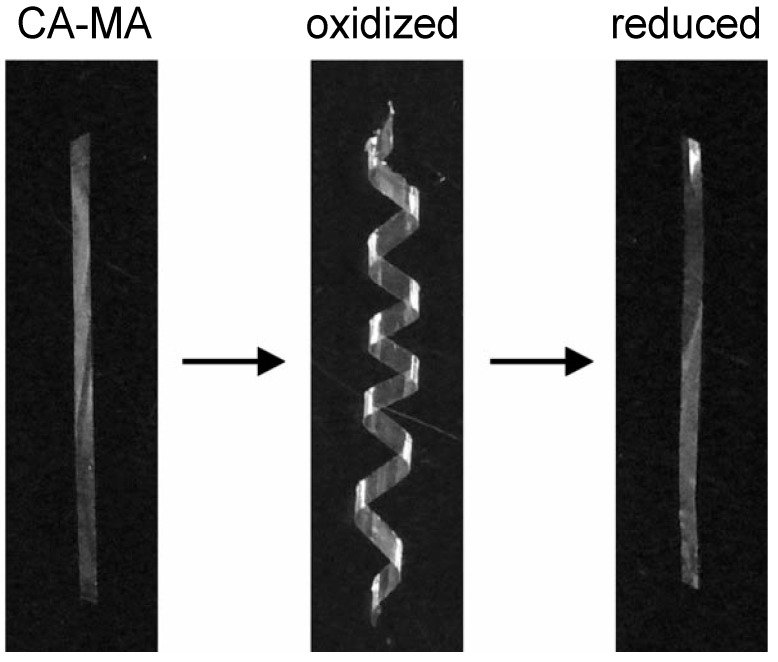
Shape memory–recovery behavior of a *ca*. 6 µm-thick CA-MA film. Reprinted with permission from ref. [34]. Copyright 2007 American Chemical Society.

Cellulose derivatization does not always involve simple organic moieties. Aoki *et al.* have prepared cellulose derivatives containing a reversible cross-linkable mercapto group by esterification of CA with mercaptoacetic acid (MA) to generate permanent wave products for hair [34]. The CA–MA products showed a shape memory–recovery behavior in film (Figure 10) and fiber forms through adequate redox treatments because of the keratin fiber-like reversible association–dissociation of the mercapto cross-links. Dimethyl sulfoxide (DMSO) acted as an oxidant while 2-mercaptoethanol or ammonium mercaptoacetic acid served as reducing reagents. The progress of the redox reactions was monitored by using a confocal depth scanning technique in Raman spectroscopy. The cross-linking effect on CA-MA film thermal and viscoelastic properties was also estimated by DSC and DMA. The loss tan δ peak alternately declined and recovered in the *T*_g_ region according to the repeated redox treatments, consistent with a reversible variation in viscoelasticity resulting from S–S cross-linking and cleavage on oxidation and reduction, respectively. These functional CA–MA derivatives are expected to find application as shape memory-recovery materials.

## 4. Blends of Simple Derivatives with Synthetic Polymers

As discussed above, side chains introduced into cellulosic molecules by derivatization generally play an effective role as “internal” plasticizers while “external” plasticizers usually facilitate the thermal molding of commodity-type cellulose derivatives. However, the more convenient “external” plasticization using low-molecular-weight substances typically leads to fume generation problems during thermal molding and plasticizer bleed-out over long-term use. The mixing of flexible higher-molecular-weight polymers as plasticizers offers an effective alternative against these problems. However, one cannot assume that any two polymers necessarily form a homogeneous blend [35]. Indeed, immiscibility occurs so frequently that miscibility/compatibility should be treated as an exception to the rule and is only observed under a precisely defined set of conditions, such as the existence of specific intermolecular interactions. This phenomenon can be rationalized by an equation developed from the thermodynamic description first proposed independently by Huggins and Flory in 1941 for a binary polymer mixture:

(2) $$\Delta G_{mix}=RT\left(\frac{V_{mix}}{V_{0}}\right)\left(\frac{\phi_{A}}{n_{A}}\ln\phi_{A}+\frac{\phi_{B}}{n_{B}}\ln\phi_{B}+\chi_{AB}\phi_{A}\phi_{B}\right)$$

where Δ*G*_mix_ is the change in Gibbs free energy upon mixing, *V*_mix_ is the volume of the mixture, *V*_0_ is the molar volume of the component, ϕ is the volume fraction, *n* is the degree of polymerization, *R* is the gas constant, and *T* is the temperature. Indices A and B represent the two separate polymers in the mixture. Polymer-polymer miscibility is therefore clearly determined by a delicate balance of enthalpic and entropic forces, where the entropic contribution is significantly smaller than in small molecule liquid mixtures (solutions). Indeed, the combinatorial entropy term, which is highly responsible for miscibility in solvents and polymer solutions, is particularly small in high-molecular-weight polymer blends because of a large degree of polymerization *n*. Therefore, polymer–polymer miscibility mainly depends on the negative heat of mixing, which results from electrostatic interactions, hydrogen bonding, dipole-dipole interactions, and dispersion forces between macromolecules. Polymer-polymer miscibility is commonly estimated by DSC determination of *T*_g_ for the blends. If a binary polymer system exhibits a single glass transition between the *T*_g_ values of individual components and its *T*_g_ shows a composition-dependent shift, the system is considered highly miscible on the *T*_g_-detection scale, which is usually assumed to be smaller than a couple of tens of nanometers [30].

### 4.1. Blend with N-Vinyl Pyrrolidone-Based Polymers

**Figure 11:**
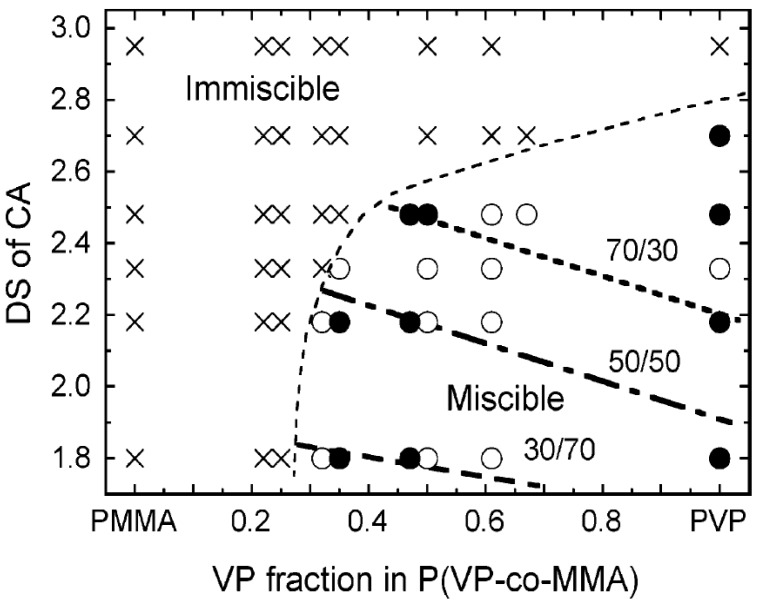
Miscibility map of the CA/P(VP-*co*-MMA) blend. Symbols indicate whether a given CA/P(VP-*co*-MMA) blend is miscible (○ and ●) or immiscible (×). Closed circles represent the blend series used in the subsequent molecular orientation study. Three lines, designated as 70/30, 50/50, and 30/70, represent critical blend compositions. Miscible polymer combinations fitting the respective lines produce a birefringence-free material at the indicated critical composition. Reprinted with permission from ref. [40]. Copyright 2007 American Chemical Society.

Despite its growing use as an industrial product, CA shows a high *T*_g_, limiting its thermal processability. Poly(*N*-vinyl pyrrolidone) (PVP) and its copolymers form miscible blends with CA and other cellulose ester (CE) derivatives by hydrogen bonding between the CE residual hydroxyl groups and the *N*-vinyl pyrrolidone (VP) carbonyl groups [36,37,38,39,40]. Ohno and Nishio characterized the miscibility of CA blends involving a random copolymer comprising VP and methyl methacrylate (MMA) units (P(VP-*co*-MMA)) in detail by DSC determination of *T*_g_ [37]. Poly(methyl methacrylate) (PMMA) plays an important role in optical and medical materials development because of its unique performance and biocompatibility. Figure 11 displays a miscibility map for the CA/P(VP-*co*-MMA) system as a function of the DS of CA and VP composition in P(VP-*co*-MMA). CA form fully homogeneous blends with P(VP-*co*-MMA) on a scale of a few nanometers when the DS of CA is below 2.75 and the VP fraction exceeds 30 mol % in the copolymer.

In general, the miscibility of CEs with VP-containing vinyl (co)polymer largely depends on a small difference in acyl substituent side-chain length (carbon number, *m*) in the employed CE, as suggested by a comparative study of blend miscibility involving cellulose propionate (CP) and butyrate (CB) in addition to CA [41,42]. The critical DS values required to obtain miscible blends of CP (2.65) and CB (2.50) with PVP were appreciably lower than for CA because the frequency of hydrogen-bonding interactions was reduced by steric hindrance from the bulkier propionyl or butyryl substituents. Unlike the situation in the CA series, high-substituted CPs (DS > 2.65) and CBs (DS > 2.5) make a miscible pair with some of the copolymers (not rich in VP) despite no contribution of the hydrogen-bonding interaction. This unique miscibility behavior, *i.e.*, advent of a miscibility window, was attributed to CE-copolymer attraction driven by repulsion between monomer units in the vinyl copolymer.

### 4.2. Blends with Aliphatic Polyesters

**Figure 12:**
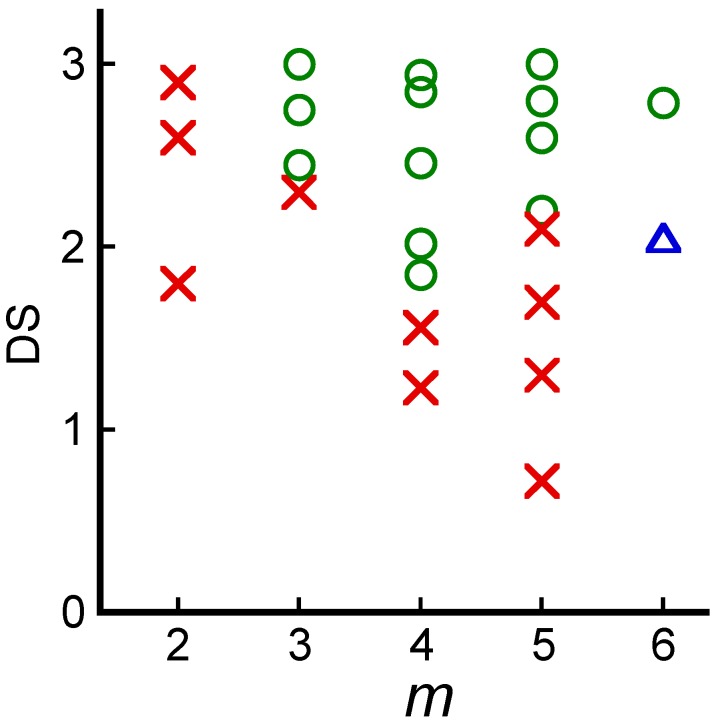
Miscibility map of different CE/PCL blends as a function of side-chain carbon number (*m*) and acyl DS. Symbols indicate whether a given CE/PCL blend is miscible (circle), immiscible (cross), or partially miscible (triangle). Reproduced with permission from ref. [45]. Copyright 2009 Elsevier Ltd.

A derivatization process, such as acylation with straight-chain fatty acids, can produce miscible blends of originally hydrophilic polysaccharides and synthetic hydrophobic macromolecules. A strong correlation has been reported between the primary structure of CE derivatives bearing various normal acyl side groups comprising *m* carbons and their miscibility with the biodegradable aliphatic polyester poly(ε-caprolactone) (PCL) [43,44]. The thermal behavior of the binary polymer blends observed by DSC clearly explained miscibility in terms of *m* and DS. Any CE derivatives showed good miscibility with PCL for *m* = 3–5 and DS ≥ 2.2 (Figure 12). In particular, butyrate (*m* = 4) exhibited a comparatively low DS of 1.9. However, most commodity-type CA (*m* = 2) was immiscible with PCL, even in a highly acetylated state. Cellulose caproate (*m* = 6) and enanthate (*m* = 7), which presented moderately long side-chains, displayed a relatively low degree of miscibility with PCL.

**Figure 13:**
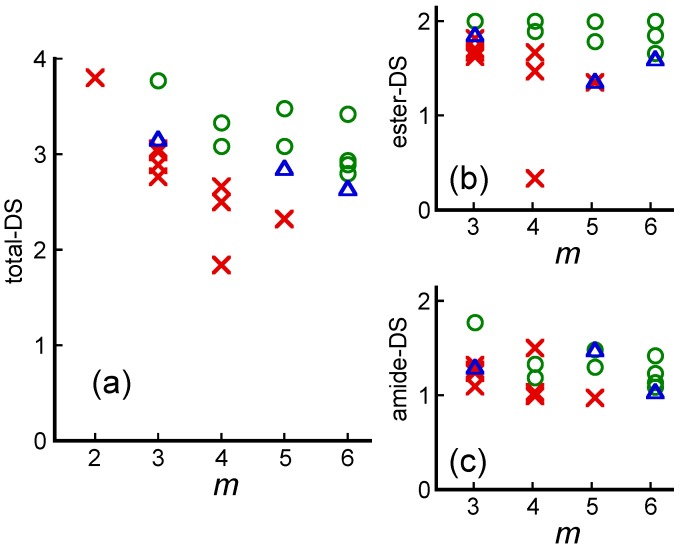
Miscibility maps of different Acyl-Ch/PCL blends as a function of side-chain carbon number *m* and substitution parameters: (**a**) total-DS; (**b**) ester-DS; and (**c**) amide-DS. Symbols indicate whether a given Acyl-Ch/PCL blend is miscible (circle), immiscible (cross), or partially miscible (triangle). Reproduced with permission from ref. [45]. Copyright 2009 Elsevier Ltd.

Teramoto *et al.* have conducted a similar investigation with chitin [45]. They synthesized acyl chitin (Acyl-Ch) derivatives containing a normal acyl group (*m* = 2–6) at different DS through a homogeneous reaction between crab-shell chitin and acyl chlorides in DMAc–LiCl solution. NMR analysis quantitatively demonstrated that the acylations occurred at C3/C6 hydroxy protons and C2 amino proton(s), which differs from the chitin diester synthesis mentioned in Section 2.2. It was therefore possible to determine the separate amide-DS and ester-DS values as well as their sum (total-DS). Figure 13 shows the estimated miscibility of Acyl-Ch/PCL blends as a function of *m* and their respective DS value. The critical total-DS (total-DS_cr_) value for achieving miscibility decreased with increasing *m* but generally surpassed the critical ester-DS for CE/PCL systems (Figure 12) for the same *m* value. In addition, miscibility increased with increasing ester-DS (Figure 13b) but lower correlation was observed between miscibility and amide-DS (Figure 13c).

Polymer *T*_g_ is directly related to their cooperative segmental motion [35], which involves 15 to 30 statistical segments. Such a cooperative motion requires sufficient thermal energy to ensure suitable ease of rotation about main chain bonds and overcome local bonding. The use of *T*_g_ to determine polymer-polymer miscibility is based on the idea that a singular *T*_g_ value indicates a domain size smaller than a few tens of nanometers. However, if one component is crystalline, such as PCL, its crystallinity is likely to regress upon addition of a second component (e.g., CE or Acyl-Ch).

^13^C cross-polarization magic angle-spinning (CP/MAS) NMR provides information on interactions between constituent polymers at the molecular level (≤1 nm). If two different polymers mutually interact at the molecular level in a binary blend, the electron density around carbon atoms bearing the interacting groups becomes perturbed. This significantly changes the ^13^C-NMR chemical shift and/or signal shape of the blend compared with those of the individual polymers. However, these spectroscopic measurements do not provide any evidence regarding specific intermolecular interactions between polysaccharide derivatives and PCL molecules in the blends.

**Figure 14:**
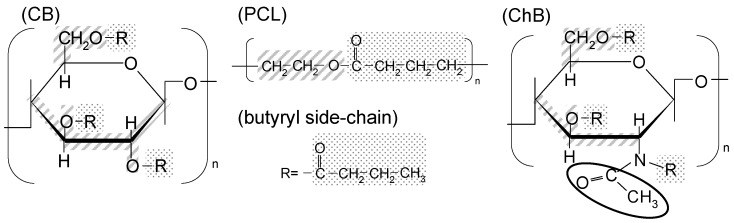
Schematic representation showing the structural similarity between the PCL repeating unit and the cellulose (CB) and chitin butyrate (ChB) butyryl side groups. Reproduced with permission from ref. [45]. Copyright 2009 Elsevier Ltd.

To determine whether miscibility was achieved in a series of polymer blends, the researchers inferred a possible contribution of dipole-dipole interactions between PCL carbonyl groups and CE and Acyl-Ch side groups. Structural affinity between acyl side groups and PCL repeating units was also considered as a potentially crucial factor to miscibility. For cellulose (CB) and chitin butyrates (ChB) (Figure 14), the butyryl side group is structurally identical with the PCL repeating unit if the glucopyranose carbon atoms are taken into account. On the other hand, in the Acyl-Ch series, the *N*-acyl substitution at the C2 position cannot improve this affinity contrary to esterification at C3/C6 positions. Therefore, higher total-DS_cr_ values are required to ensure miscibility. Actually, the miscibility increased when ester-DS increased (Figure 13b) but the correlation between miscibility and amide-DS was lower (Figure 13c). The presented information strongly suggests a direct correlation between the primary structure of the polysaccharide derivatives and their miscibility with the hydrophobic polymer.

**Figure 15:**
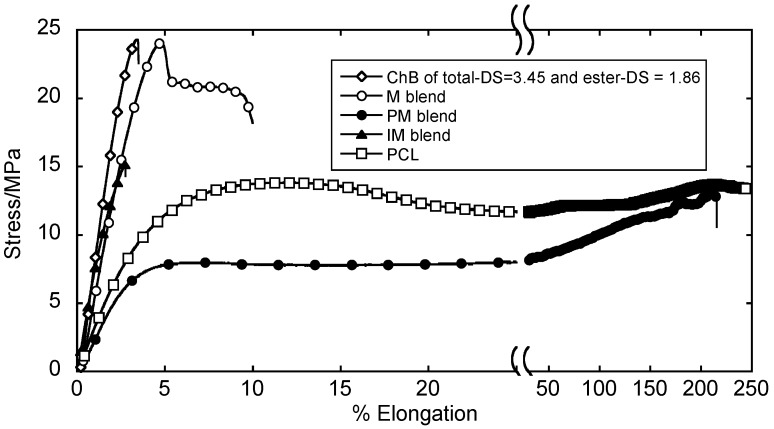
Stress–strain curves of ChB (total-DS = 3.45; ester-DS = 1.86) and PCL and their IM, PM, and M blends (ChB/PCL = 50/50 (*w*/*w*)). Reproduced with permission from ref. [46]. Copyright 2014 Elsevier Ltd.

**Figure 16:**
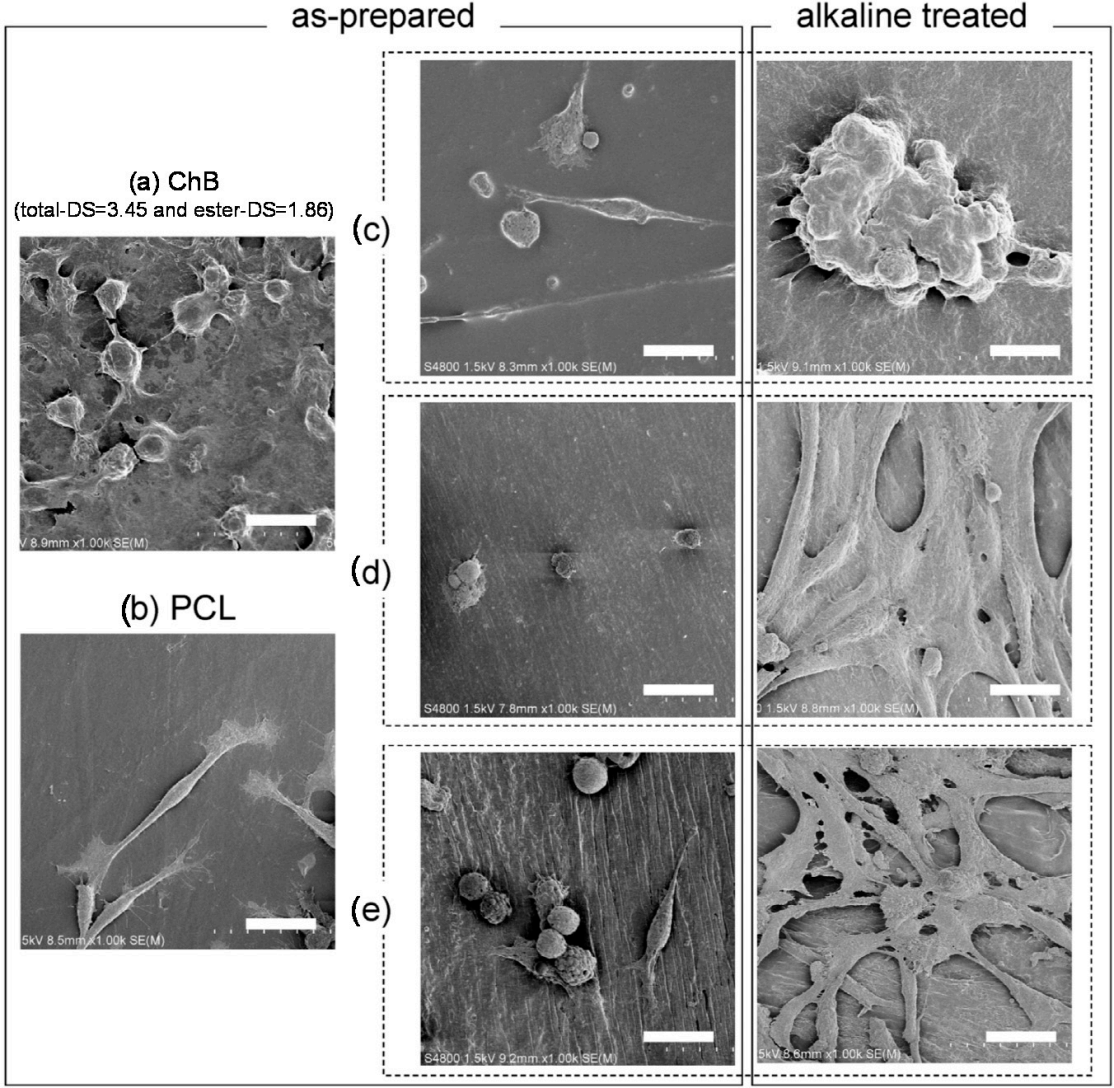
Field emission scanning electron microscope (FE-SEM) images of cell growth on films of (**a**) ChB (total-DS = 3.45; ester-DS = 1.86); (**b**) PCL; and (**c**) M; (**d**) PM, and (**e**) IM blends (original ChB/PCL weight ratio = 50/50). Images (**c**)–(**e**) include those of alkaline treated films. Scale bars denote 20 µm. Reproduced with permission from ref. [46]. Copyright 2014 Elsevier Ltd.

The degree of miscibility significantly affects material performances, such as mechanical strength and cytocompatibility [46]. Teramoto *et al.* have fabricated thermoplastic transparent films from ChB/PCL blends [46]. Even though the mechanical properties of the immiscible (IM) blends did not improve, the ductility of ChB increased in miscible (M) and partially miscible systems (PM), making these blends highly processable (Figure 15). DSC analysis showed that PM blends slightly shifted the *T*_g_ of the ChB component but enhanced that of PCL. These blends exhibited extremely high ductility (tensile strength ≈15 MPa; elongation at break >200%), which may result from their ideal balance between PCL microcrystallite size and dispersion as well as the moderate domain size (20–50 nm) of the amorphous blend components. These blend films were subsequently subjected to alkaline hydrolysis (2 M NaOH/37 °C/48 h) to enhance their surface hydrophilicity and cell accessibility. Attenuated total reflection (ATR)-FTIR spectroscopy results revealed that the PCL component and ChB ester side chains were selectively removed from the hydrolyzed PM and IM film surface domains, causing a localization in chitin concentration in these surface domains. L929 fibroblast cells adhered well and prolifically distributed on PM film surfaces (Figure 16). Therefore, these materials exhibit great potential for use in tissue engineering as thermoplastic cell scaffolds. These scaffolds adopt various three-dimensional forms under degrees of miscibility selected according to adequate DS and post treatment variation.

## 5. Graft Copolymers

Graft copolymerization offers a practical way to modify cellulosic molecules and alter woody materials surface properties. It may also prove useful to improve some original properties of polysaccharides and promote novel bulk functions in the copolymer products. In contrast to extensive structural and physical studies for block copolymer and polymer blends, systematic investigations on graft copolymer morphology, thermal transition behavior, and mechanical properties had been restricted to few systems including synthetic/synthetic polymer pairs in the past century [47]. Considerable efforts have been deployed to establish the relationship between copolymer compositions and properties in detail and functionalize cellulosic graft copolymers.

In contrast to unmodified cellulose, cellulose derivatives exhibit a relatively good but DS-dependent solubility in some organic solvents, making reaction possible in an appropriate homogeneous system. For DS < 2.5, CA is usually biodegradable [48,49] and its functionalization may benefit many chemical industries as well as agroindustrial, sanitary, and bio-related fields. Graft copolymerization provides an alternative to overcome above-mentioned problems related to low-molecular-weight “external” plasticizers. Aliphatic hydroxy acids or cyclic esters are attractive monomers for grafting CA for environmental conformity.

Higher-molecular-weight poly(hydroxy alkanoate)s (PHAs) are biodegradable polymers synthesized by polycondensation of hydroxy acids [50] and ring-opening polymerization of cyclic esters, such as lactides and lactones. The ring-opening polymerization is initiated efficiently using hydroxo-initiators [51] involving CAs [52,53,54] and CEs except the esters substituted completely such as cellulose triacetate.

**Figure 17:**
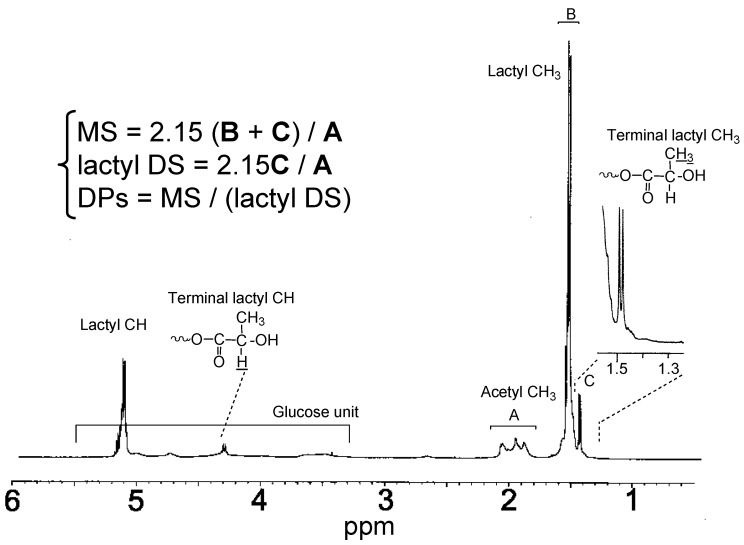
^1^H-NMR spectrum of a CA-*g*-PLA for which acetyl-DS = 2.15, MS = 7.86, DPs = 8.93, and lactyl DS = 0.88. Reproduced with permission from ref. [55]. Copyright 2003 Elsevier Science Ltd.

Molar substitution (MS) and oxyalkanoyl DS, defined as the average number of introduced oxyalkanoyl units and that of hydroxyl groups substituted for oxyalkanoyl units per CA anhydroglucose residue, respectively, are important parameters for CE-*graft*-PHAs graft copolymer characterization. They are typically determined by ^1^H NMR, as illustrated in Figure 17 for CA-*graft*-poly(lactic acid) (CA-*g*-PLA), giving the average degree of polymerization (DPs) of the PHA side chain as MS/(oxyalkanoyl DS). Equations used to calculate MS, oxyalkanoyl (lactyl) DS, and DPs are shown in Figure 17. Here, CE*_x_*-*g*-PHA*_y_* corresponds to CE-*g*-PHA copolymer for which ester-DS = *x* and oxyalkanoyl MS = *y*.

### 5.1. Synthesis of Biodegradable Cellulosic Graft Copolymers and Formulation of Basic Physical Properties

**Figure 18:**
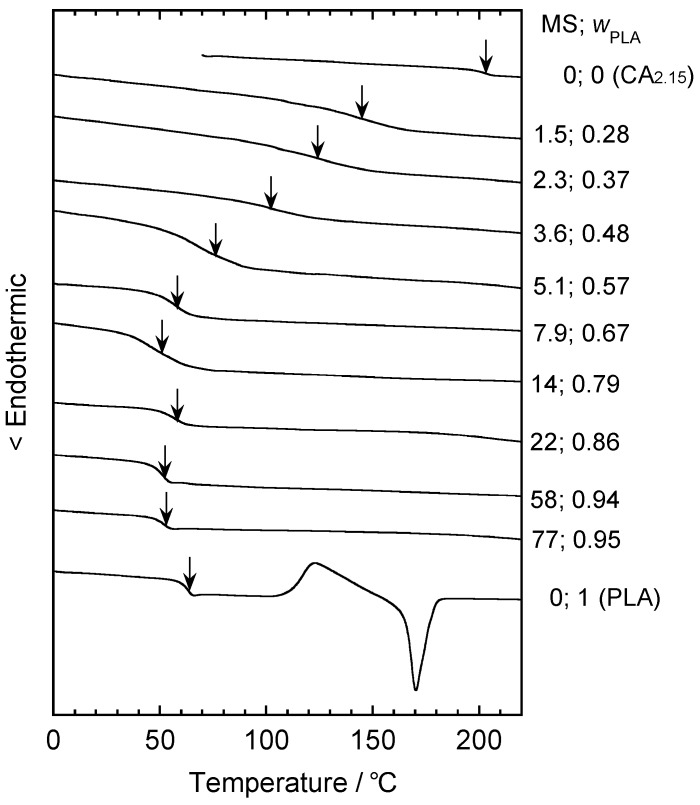
DSC thermograms of CA_2.15_, PLA, and CA_2.15_-*g*-PLAs obtained at various MS values during in the second heating scan. Arrows mark the *T*_g_ positions taken as the midpoints of the heat flow discontinuity. Reproduced with permission from ref. [55]. Copyright 2003 Elsevier Science Ltd.

**Figure 19:**
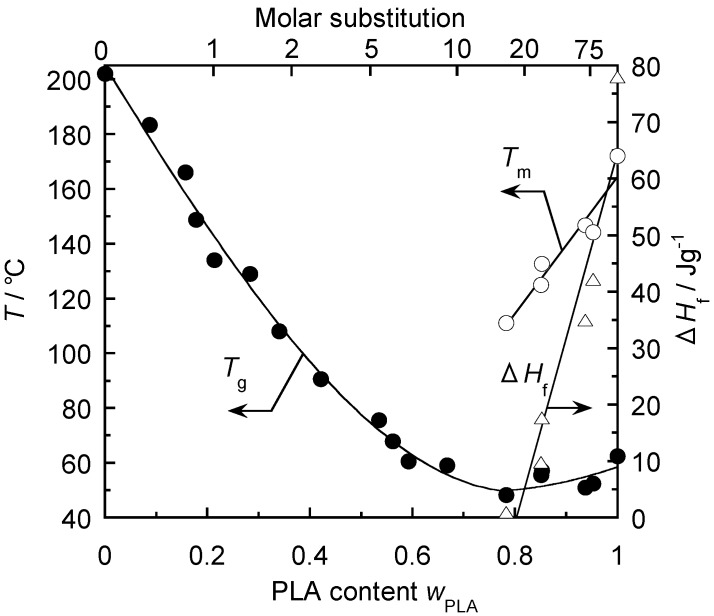
Composition dependence of the thermal transition parameters *T*_g_, *T*_m_, and *ΔH*_f_, estimated by DSC for CA_2.15_-*g*-PLA samples. *T*_m_ and *ΔH*_f_ were obtained in the first run while *T*_g_ was evaluated in the second heating scan. Reproduced with permission from ref. [55]. Copyright 2003 Elsevier Science Ltd.

**Figure 20:**
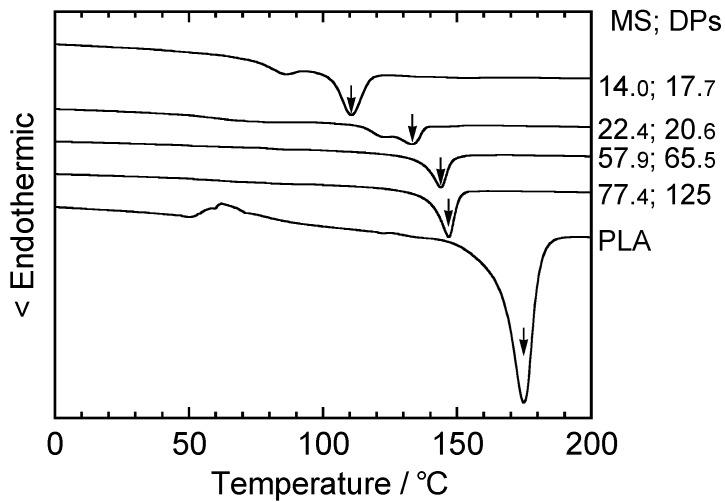
DSC thermograms of CA_2.15_-*g*-PLA samples at different MS values. Arrows indicate *T*_m_ positions taken as endothermic peak maxima. Reproduced with permission from ref. [55]. Copyright 2003 Elsevier Science Ltd.

Teramoto and Nishio prepared CA_2.15_-*g*-PLAs with a wide range of composition by three grafting methods, including (1) copolycondensation of lactic acid, (2) ring-opening copolymerization of l-lactide in DMSO, and (3) bulk ring-opening copolymerization similar to method (2) but without DMSO [55]. DSC measurements revealed that all copolymer products exhibited a single *T*_g_ (Figure 18). When lactyl MS increased to 8, *T*_g_ decreased sharply from 202 °C, corresponding to that of original CA, to *ca.* 60 °C, approximating that of plain PLA (Figure 19). When MS exceeded 14, the as-prepared graft copolymers formed a PLA side-chain crystalline phase (Figure 20). Tensile measurements of melt-quenched CA_2.15_-*g*-PLA film sheets conducted at 80 °C–100 °C showed that their stretchability increased drastically with increasing PLA content and, for MS ≥ 14, the elongation at rupture reached a maximum of *ca*. 2000%.

Beside CA_2.15_-*g*-PLA, CA-*g*-PHAs were subsequently synthesized by applying earlier chemical polymerization examples involving (R,S)-β-butyrolactone (BL), δ-valerolactone (VL), and ε-caprolactone (CL) [56]. To enhance the diversity of molecular architectures using this graft series, acetyl-DS was varied for the starting polymer CA to control the intramolecular density of grafts. The resulting CA-*g*-PHAs were characterized to establish a relationship between molecular architecture and thermal transition behavior in terms of semi-empirical equations proposed for polymer blends and comb-like polymers. Fox and Gordon–Taylor equations were assumed unsuitable for depicting the composition dependence of observed *T*_g_ for this graft copolymer series. However, if the molecular weight dependence of the side-chain component *T*_g_ is taken into account, the composition dependence of *T*_g_ obeys a simple mixing rule between CA_2.15_ and PLA ingredients for CA_2.15_-*g*-PLAs although the CA-*g*-PHA copolymers consist of originally immiscible components in this case. On the other hand, the *T*_g_ expression proposed for the comb-like polymer model [57] may provide an alternative depiction of the composition dependence of *T*_g_ for CA graft copolymers exhibiting acetyl-DS ranging from 1.75 to 2.15 despite some limits in its application to the assessment of multiple data-dependent parameters. However, the observed *T*_g_ clearly deviated from the calculated curve for the CA graft series at acetyl-DS values of 2.45 and 2.98, suggesting a possible phase separation of the two copolymer components. This comb-like polymer approximation would not be applicable to cases where the molecular weight of the backbone segments between two adjacent grafting positions is actually too high.

### 5.2. Thermal Treatment Effect on the Development of Supramolecular Structures

**Figure 21:**
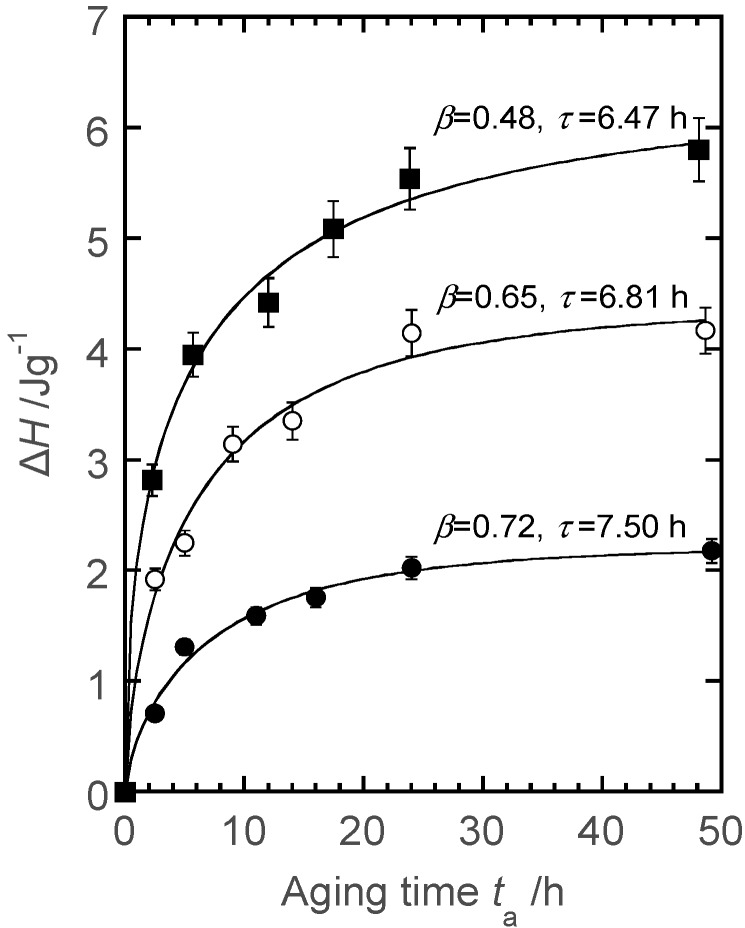
Time evolution of relaxation enthalpy ∆*H* estimated for PLLA (■) and CA_2.15_-*g*-PLLAs at MS values of 4.7 (●) and 22 (○). The aging temperature amounted to 50 °C. Reproduced with permission from ref. [58]. Copyright 2004 American Chemical Society.

The development of supramolecular structures of CA_2.15_-*g*-poly(l-lactide) (PLLA) was investigated via physical aging and crystallization experiments under isothermal conditions [58]. For copolymers presenting MS values of 4.7 and 22 aged at different temperatures below or close to their *T*_g_s (77 °C and 55 °C, respectively) for a definite time period, an optimum temperature of 50 °C was obtained to detect the largest enthalpy relaxation *∆H* by DSC. The time evolution of *∆H* at 50 °C was analyzed satisfactorily using the Kohlrausch–Williams–Watts stretched exponential function (Figure 21). Overall relaxation times (τ) were somewhat longer for the graft copolymers than for pristine PLLA. The regression analysis provided an exponent β characterizing the relaxation time distribution in addition to fairly large values for the aged copolymers compared with the relaxation behavior of conventional amorphous polymers and PLLA homopolymer. The anchoring of PLLA chains, which mainly contribute to the observed relaxation, onto the semirigid CA_2.15_ backbone may significantly restrict their motions, resulting in a narrower distribution of the relaxation enthalpy.

**Figure 22:**
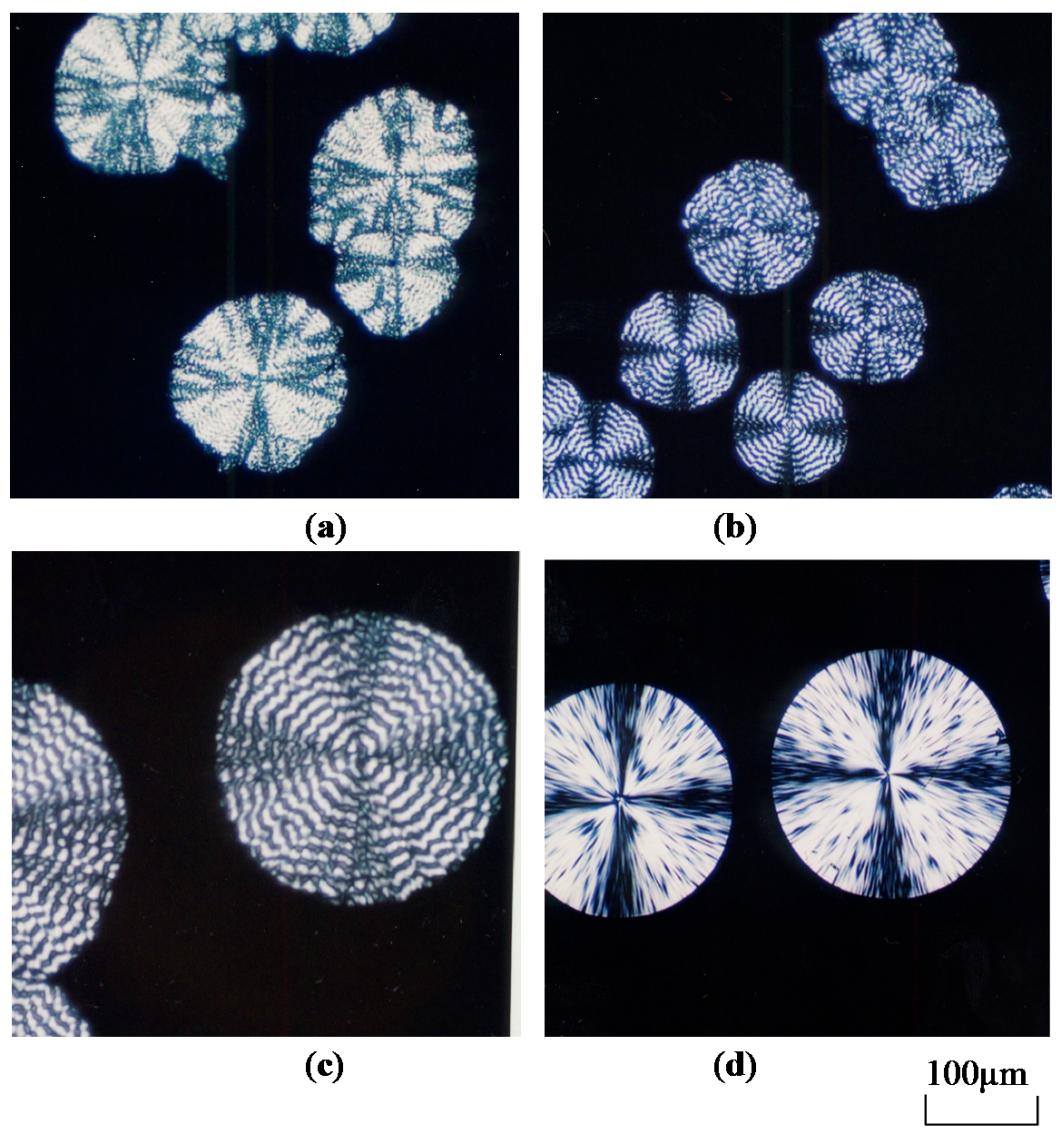
Polarized optical micrographs of typical spherulites observed for CA_2.15_-*g*-PLLAs and plain PLLAs: (**a**) MS = 22 at *T*_ic_ = 107 °C and *t* = 4000 min; (**b**) MS = 58 at *T*_ic_ = 119 °C and *t* = 365 min; (**c**) MS = 77 at *T*_ic_ = 110 °C and *t* = 300 min; (**d**) PLLA at *T*_ic_ = 130 °C and *t* = 30 min. *T*_ic_ is the isothermal crystallization temperature and *t* is the elapsed time. Reproduced with permission from ref. [58]. Copyright 2004 American Chemical Society.

An isothermal crystallization experiment conducted using a polarized optical microscope (POM) showed that CA_2.15_-*g*-PLLAs underwent spherulite growth for MS values ranging from 22 to 77 (Figure 22) [53]. The optical texture comprised extinction rings, forming a band pattern of concentric circles, which had never been reported for plain PLLA and other graft copolymers. When MS increased, the texture became distorted and lost contrast while the growth rate dropped dramatically. Therefore, PLLA-rich graft copolymers shared common features with some crystalline polymer blends containing low concentrations of compatible polymer diluents regarding spherulite texture and growth rate. The slower growth kinetics caused by the anchoring effect was analyzed quantitatively to estimate the surface free energy σ_e_ of the folded PLLA lamellar crystals constituting the respective spherulites. This analysis was performed using a folded-chain crystallization formula expanded for a binary system composed of a miscible crystalline/amorphous polymer pair. Despite little MS-related systematic changes, the dependence of σ_e_ on the copolymer composition was interpretable through a convincing discussion on the stability parameter ϕ estimated from melting point measurements.

### 5.3. Enzymatic Hydrolysis and Surface Morphological Characterization

Two compositions of the CA_2.15_-*g*-PLLA series (MS = 4.7 (L) and 22 (H)) were subjected to enzymatic hydrolysis using Proteinase K [59]. A q-series of tested film specimens were only quenched from the molten state, and others were further annealed above and below their *T*_g_s. Weight loss data recorded as a function of elapsed time revealed that the hydrolysis rate decreased as a result of the graft modification itself and the low PLLA content unless PLLA crystallinity developed. The adjacent hydrophobic CA2.15 backbone may seriously hinder the enzymatic attack of the PLLA side chains. Heat treatments, followed by physical aging or partial crystallization of the originally amorphous materials, also reduced the degree of enzymatic hydrolysis. Thus, the enzymatic degradation of CA_2.15_-*g*-PLLAs was temporally controlled through variations of the molecular compositional factor and supramolecular rearrangement possibly occurring during isothermal treatments, such as tighter PLLA graft packing and free volume reduction.

**Figure 23:**
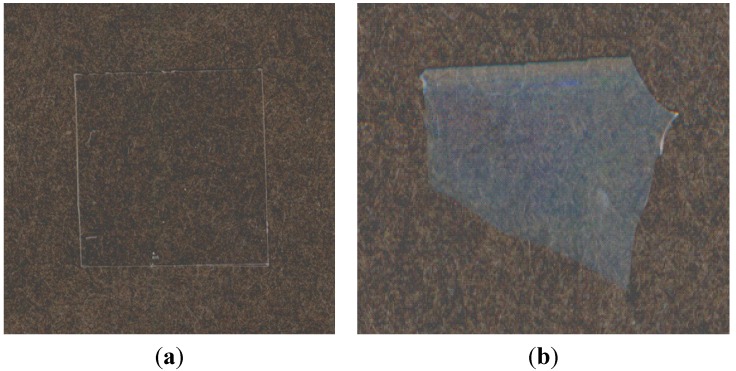
Photographs of an H-q sample (**a**) before and (**b**) after a 20-day enzymatic hydrolysis. Reproduced with permission from ref. [59]. Copyright 2004 American Chemical Society.

To determine the spatial development of the enzymatic hydrolysis, surfaces of q-series degraded copolymer films were characterized by atomic force microscopy (AFM) and ATR-FTIR. The AFM study showed that the enzymatic hydrolysis produced a more undulated surface exhibiting several protuberances measuring several-hundred nanometers in height and a few micrometers in width. The hydrolysis generated finer and uniform protuberances on the L-q film surface while much deeper erosions, but somewhat irregular in amplitude, prevailed on the degraded H-q film surface at a higher PLLA content. ATR-FTIR measurements revealed that the hydrolyzed film surfaces selectively released lactyl units and absorption intensities were consistent with the AFM results. The degraded film specimens often displayed an iridescent color (Figure 23). This may be attributed to the interference of visible light wavelengths resulting from the diffuse reflection between the protuberances formed on film surfaces, which is virtually interpretable as a thin-layered gradation of refractive indices near the film surface. The complementary observations of after-effects of the enzymatic hydrolysis for the graft copolymers embody a conception of “spatiotemporally controlled degradation” for design of novel polymeric materials endowed with multi-functionalities, which involves degradation rate regulation, surface modification, and ensuing physical property improvement.

### 5.4. Molecular and Segmental Dynamics Characterized by Relaxation Analysis

Molecular and segmental dynamics of CA-*g*-PCL and CB-*g*-PCL components were investigated by dynamic mechanical, dielectric, and nuclear magnetic relaxation measurements in addition to their mixing scale [60].

Different relaxation processes originating from the molecular motions of the relevant structural units were detected by DMA for CA-*g*-PCL and CB-*g*-PCL series. Molecular mobility as a whole was enhanced when the introduction rate of the flexible PCL side chains increased for the copolymer series. Both CA-*g*-PCL polymer constituents tended to undergo phase separation on a *ca*.15 nm scale because of the poor miscibility between CA trunk and PCL side chains. In contrast, CB trunk chains mixed more intimately with aliphatic polyester side chains in CB-*g*-PCLs, reflecting the high miscibility of the CB/PCL pair as shown in Figure 12. However, CB-based copolymer components tended to adopt a heterogeneous mixing state at lower MS (0.5–0.6) because the anchoring of PCL chains onto the semirigid CB trunk affects chain mobility.

Dielectric relaxation spectroscopic analysis revealed that the introduction of CL units hardly affected the chain segmental velocity of the CA trunk. The constant relaxation time distribution for the α relaxation of the CA component suggested that there was almost no correlation in chain segmental dynamics between CA and PCL components. In contrast, the CB-*g*-PCL series exhibited a higher cooperation in segmental dynamics between trunk and side chain polymers, which may stem from the original miscibility of the CB/PCL pair. This enhanced miscibility, which originates from structural affinity and dipole-dipole interactions between butyryl groups and PCL repeating units, appeared to give rise to a dynamical interaction even on a local structural scale.

**Figure 24:**
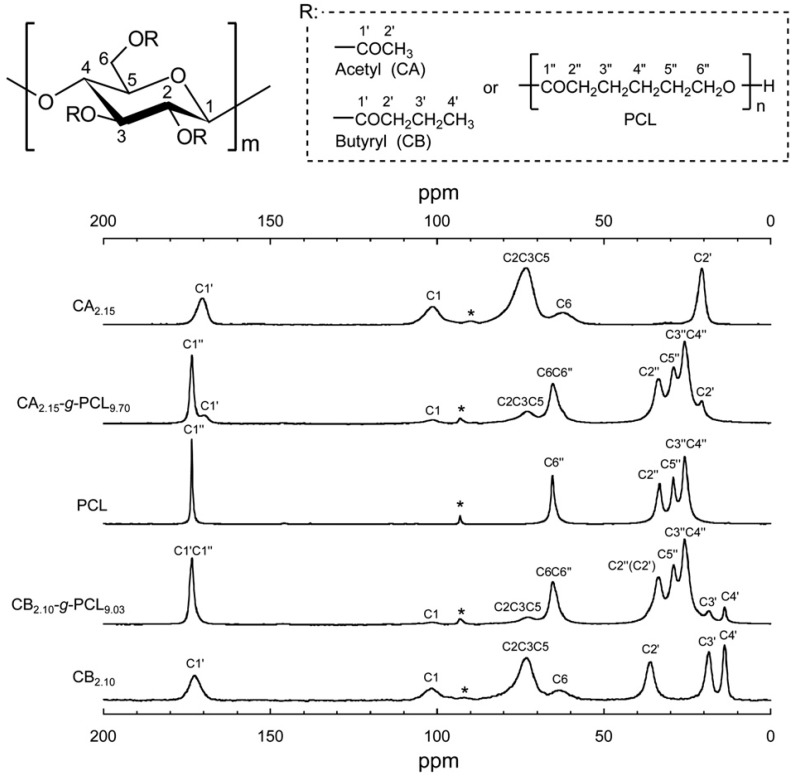
Solid-state ^13^C CP/MAS NMR spectra of CA_2.15_ (DS = 2.15), CA_2.15_-*g*-PCL_9.70_, plain PCL, CB_2.10_-*g*-PCL_9.03_, and CB_2.10_, and their peak assignments. Asterisks denote a spinning sideband overlapping with the C4 pyranose carbon resonance signal. The C2" peak for CB_2.10_-*g*-PCL_9.03_ overlaps with the low intensity peak of the butyryl substituent C2'. Reproduced with permission from ref. [60]. Copyright 2011 Elsevier Ltd.

The ^1^H spin-lattice relaxation time in the rotating frame (*T*_1ρ_^H^) determined by solid-state NMR (Figure 24) for specific carbons in a multicomponent polymer system provides an estimate of mixing homogeneity at the ^1^H spin-diffusion length scale (2–4 nm). In general, *T*_1ρ_^H^ values are obtained by fitting the decaying carbon resonance intensity with the following single-exponential equation:

(3) $$M\left(t\right)=M\left(0\right)\text{exp}(-t/T_{1\rho }^{H})$$

where *M*(*t*) is the magnetization intensity observed as a function of the spin-locking time *t*. In practice, $T_{1\rho }^{H}$ is determined from the slope of the plot of ln[*M*(*t*)/*M*(0)] against *t*. Data confirmed that molecular mobility as a whole was promoted by the introduction of flexible PCL side chains for MS < 4. The promoted mobility is an alternative expression of the internal plasticization effect. However, for graft copolymers (MS > 7) containing a distinct crystalline phase expected to show a slower magnetization decay, the logarithmic *M*(*t*) data hardly fitted a single straight line. In this case, the normalized *M*(*t*) was simulated using a bi-exponential function involving two relaxation times as [61]:

(4) $$M(t)/M(0)=x_{f}\text{exp}(-t/T_{1\rho ,\;fast}^{H})x_{s}\text{exp}(-t/T_{1\rho ,slow}^{H})$$

where $T_{1\rho ,fast}^{H}$ and $T_{1\rho ,slow}^{H}$ represent $T_{1\rho }^{H}$ values for flexible (faster decay) and rigid (slower decay) components, respectively, and *x*_f_ and *x*_s_ are the corresponding fractions.

**Figure 25:**
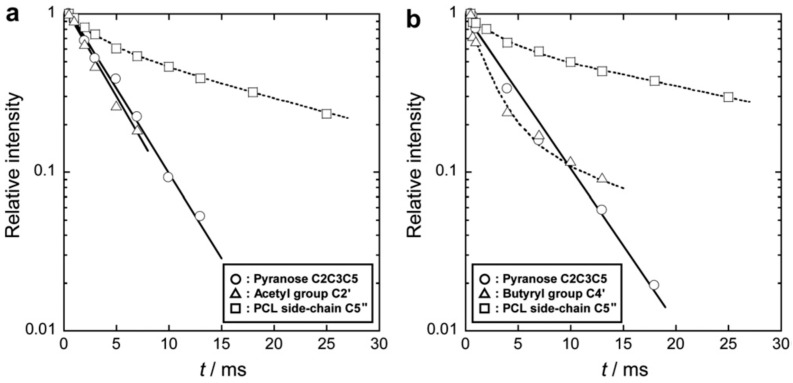
Semilogarithmic plots of ^13^C resonance intensity decay as a function of spin-locking time *t* for (**a**) CA_2.15_-*g*-PCL_9.70_ and (**b**) CB_2.10_-*g*-PCL_9.03_ films. Straight lines represent fits to a single-exponential function (see Equation (3)) for the acetyl C2' in CA_2.15_-*g*-PCL_9.70_ and for the pyranose C2/C3/C5 in CA_2.15_-*g*-PCL_9.70_ and CB_2.10_-*g*-PCL_9.03_. Dashed-line curves correspond to fits to a double exponential function (see Equation (4)), where *x*_f_ = 0.34 and *x*_s_ = 0.66 for the PCL side-chain C5" in CA_2.15_-*g*-PCL_9.70_, *x*_f_ = 0.82 and *x*_s_ = 0.18 for the butyryl C4' in CB_2.10_-*g*-PCL_9.03_, and *x*_f_ = 0.32 and *x*_s_ = 0.68 for the PCL side-chain C5" in CB_2.10_-*g*-PCL_9.03_. Reproduced with permission from ref. [60]. Copyright 2011 Elsevier Ltd.

When CA acted as the trunk, the magnetization decay of the acetyl C2' resonance yielded a $T_{1\rho }^{H}$ value that almost coincided with that of another $T_{1\rho }^{H}$ obtained by monitoring the pyranose C2/C3/C5 signal, as demonstrated for CA_2.15_-*g*-PCL_9.70_ in Figure 25a. $T_{1\rho }^{H}$ data for CEs, PCL, and CE-*g*-PCLs are listed in Table 1. These values corresponding to the CA component were intermediate between $T_{1\rho ,fast}^{H}$ and $T_{1\rho ,slow}^{H}$ values for the crystallizable PCL. These observations suggest that the acetyl group is firmly restrained to the cellulose backbone and the molecular mobility of the unified CA trunk is somewhat restricted by the contiguous PCL crystalline domains in PCL-rich CA-based copolymers. On the other hand, the decay of the butyryl C4' signal was characterized by two different $T_{1\rho }^{H}$ values while that of the skeletal C2/C3/C5 signal provided a single $T_{1\rho }^{H}$ for CB-based copolymers exhibiting MS values exceeding 7. The representative behavior of CB_2.10_-*g*-PCL_9.03_ is shown in Figure 25b. Shorter and longer $T_{1\rho }^{H}$ values obtained for the butyryl C4' signal decay were associated with amorphous and ordered phases, respectively, by comparison with other $T_{1\rho }^{H}$ data for CB-*g*-PCL samples displaying the same butyryl DS. This observation indicates that the butyryl substituent may be free from restraint to the cellulose backbone and partly intrude into the PCL lamellar crystal surface, in agreement with crystallization kinetic studies [62].

**Table 1:** $T_{1\rho }^{H}$ data for CA, CB, PCL, and their graft copolymers. Reproduced with permission from ref. [55]. Copyright 2011 Elsevier Ltd.

Samples	$T_{1\rho }^{H}$/ms			
	CA or CB Component		PCL Component	
	Pyranose C2C3C5	Acetyl C2' or Butyryl C4'	C3''C4''	C5''
CA_2.15_	13.7	13.9	–	–
CA_2.15_-*g*-PCL_0.27_	12.9	13.3	12.8	12.3
CA_2.15_-*g*-PCL_0.87_	8.46	7.87	8.02	8.30
CA_2.15_-*g*-PCL_1.30_	6.99	7.11	4.72	3.72
CA_2.15_-*g*-PCL_2.50_	2.79	2.74	2.20	1.71
CA_2.15_-*g*-PCL_9.70_	4.05	3.79	3.01 ^(a)^/24.3 ^(b)^	3.63 ^(a)^/24.6 ^(b)^
CA_2.45_	16.6	15.9	–	–
CA_2.45_-*g*-PCL_0.11_	14.0	14.4	13.6	13.2
CA_2.45_-*g*-PCL_0.22_	11.4	11.7	9.03	7.23
CA_2.45_-*g*-PCL_1.20_	6.21	5.64	4.02	4.17
CA_2.45_-*g*-PCL_2.50_	3.02	2.68	2.05	2.39
CA_2.45_-*g*-PCL_9.30_	7.49	8.31	4.10 ^(a)^/22.5 ^(b)^	4.83 ^(a)^/23.3 ^(b)^
CA_2.98_	15.7	15.4	–	–
CA_2.98_-*g*-PCL_0.22_	13.7	13.9	8.95	9.03
CA_2.98_-*g*-PCL_0.55_	12.3	14.0	7.62	6.39
CA_2.98_-*g*-PCL_2.07_	6.34	5.64	3.49	4.52
CA_2.98_-*g*-PCL_9.20_	7.09	7.87	2.15 ^(a)^/22.0 ^(b)^	1.75 ^(a)^/21.6 ^(b)^
CB_2.10_	9.20	8.64	–	–
CB_2.10_-*g*-PCL_0.16_	7.45	6.73	8.58	6.42
CB_2.10_-*g*-PCL_0.60_	6.58	6.23	6.47	5.23
CB_2.10_-*g*-PCL_2.33_	3.24	3.11	2.48	2.69
CB_2.10_-*g*-PCL_9.03_	3.94	2.02 ^(a)^/18.0 ^(b)^	3.86 ^(a)^/27.3 ^(b)^	3.00 ^(a)^/31.1 ^(b)^
CB_2.50_	7.60	7.90	–	–
CB_2.50_-*g*-PCL_0.26_	7.09	7.26	n.d.^c)^	n.d.
CB_2.50_-*g*-PCL_1.37_	3.89	3.23	3.45	n.d.
CB_2.50_-*g*-PCL_3.49_	3.26	3.50	3.96	3.55
CB_2.50_-*g*-PCL_7.42_	4.82	3.20 ^(a)^/18.3 ^(b)^	3.68 ^(a)^/29.4 ^(b)^	4.83 ^(a)^/31.6 ^(b)^
CB_2.93_	8.26	7.60	–	–
CB_2.93_-*g*-PCL_0.23_	7.73	7.67	n.d.	n.d.
CB_2.93_-*g*-PCL_0.50_	5.81	5.36	n.d.	n.d.
CB_2.93_-*g*-PCL_3.58_	3.25	2.80	3.64	3.00
CB_2.93_-*g*-PCL_12.6_	n.d. ^(c)^	3.12 ^(a)^/18.7 ^(b)^	7.20 ^(a)^/35.0 ^(b)^	7.29 ^(a)^/38.0 ^(b)^
PCL	–	–	6.29 ^(a)^/60.2 ^(b)^	7.17 ^(a)^/61.8 ^(b)^

^(a)^ $T_{1\rho ,fast}^{H}$; ^(b)^ $T_{1\rho ,slow}^{H}$; ^(c)^ undetectable.

### 5.5. Extension of Reaction Systems for Graft Copolymerization

The effective use of polysaccharide hydroxyl groups as initiators for the ring-opening polymerization of lactides and lactones to fabricate graft copolymers is expected to witness growth. Kitaoka *et al.* prepared partially substituted cellulose graft copolymers by regioselective derivatization of cellulose with l-lactide and CL in homogeneous DMAc–LiCl solution using native cellulose fiber as a starting material [63]. The resulting copolymers exhibited good solubility in common organic solvents. Enzymatic degradation of the copolymers occurred immediately upon cellulase treatment, even under mild conditions that do not hydrolyze commercial CDA. Vidéki *et al*. investigated the effect of external and internal (grafting) plasticization of CA using PCL and found that the external plasticizer was less efficient at reducing the stiffness of CA than grafted PCL chains [64]. Yuan *et al.* introduced PCL and PLLA block copolymers as grafted side chains in ethyl cellulose (EC) [65]. The EC-*g*-PCL spherulites formed sharply defined extinction rings. These researchers also fabricated thermoplastic cellulose-*g*-PLLA graft copolymers by ring-opening polymerization of l-lactide in homogeneous 1-allyl-3-methylimidazolium chloride ionic liquid solution using 4-dimethylaminopyridine as an organic catalyst and unmodified cellulose as a starting material [66]. Lönnberg *et al*. performed the ring-opening polymerization of CL and l-lactide from unmodified filter paper and paper preactivated with xyloglucan-bis(methylol)-2-methylpropanamide (XG-bis-MPA) and 2,2-bis(methylol)propionic acid (bis-MPA), respectively [67]. The bis-MPA activated paper exhibited the highest grafting efficiency, giving the most promising results regarding compatibility improvement between components and thus improving the mechanical properties. AFM observations showed that grafting produced a less-defined fibrillar structure and much smoother surface, indicating that the polymer covered the paper surface. Habibi *et al.* reported that PCL was covalently grafted onto the surface of cellulose nanocrystals suspended in dry toluene [68]. The obtained PCL-grafted cellulose nanocrystals exhibited significantly enhanced mechanical performances compared to those dispersed in PCL by solution casting. Enomoto-Rogers and Iwata synthesized di-*O*-(6-azidohexanoyl)-xylan-*graft*-PLLA coplymers by grafting propargyl-terminated PLLA onto di-*O*-(6-azidohexanoyl)-xylan (XylC6N_3_) via click chemistry [69]. DSC measurements revealed that grafted PLLA side chains played an effective role as internal plasticizers for XylC6N_3_ and native xylan.

## 6. Orientation Control

Processing methods such as stretching, spinning, and rolling are applicable to polymeric materials to produce polymer films and fibers exhibiting direction-dependent physical properties. This anisotropic behavior is at least partly attributable to the preferred orientation of molecular chains in non-crystalline regions, crystallite orientation, and, to some extent, the orientation of the supramolecular structure itself. Because a variety of thermal processing methods can be potentially employed for the thermoplasticized cellulosics mentioned above, it is expected to introduce some specific material functions based on anisotropy into the systems. It is therefore important to clarify the relationship between molecular orientation behavior and physical properties.

Controlling the optical anisotropy of CE-based materials has recently attracted increasing interest [40,70]. Specifically, CA is expected to provide a film base for optical devices used to regulate the polarization of light. Triacetyl cellulose (TAC) has been applied to liquid crystal display (LCD) panels as a protective layer for their poly(vinyl alcohol) polarizing films. However, the resulting birefringence creates imperfect optical compensation, leading to a color shift at oblique incidence and/or a reduction in black/white contrast [71]. Therefore, the selection, regulation, or compensation of linearly polarized light in these materials rests on the development of methods that control their optical properties.

**Figure 26:**
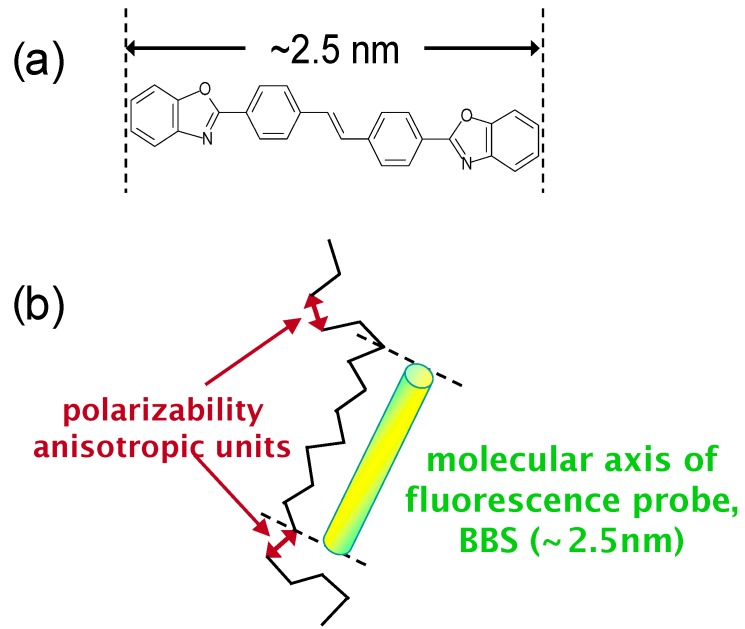
(**a**) Molecular structure of BBS and (**b**) schematic representation of the dimensional and directional differences between the polarizability anisotropic units and BBS.

The macromolecule orientation distribution is typically characterized by (i) WAXD, which only applies to crystalline polymers, (ii) dichroic infra-red or UV-visible spectroscopy and birefringence measurements, which provide secondary orientation functions, as well as (iii) fluorescence polarization measurements and laser Raman spectroscopy, which simultaneously give the statistic second and fourth moments. Interestingly, a combination of methods (ii) and (iii) enables an evaluation of the degree of orientation and the orientation distribution. This review focuses on examples that combine fluorescence polarization and birefringence (*Δn*) measurements. In fluorescence polarization measurements, the fluorescent 4,4'-bis(2-benzoxazolyl)stilbene (BBS, Figure 26a; length: *ca.* 2.5 nm) is mixed with polymer films and utilized as a probe to estimate their overall molecular orientation. Optical birefringence determines the orientation of polymer chains that display inherent anisotropy to their polarizability. Therefore, the difference between the dimensions of BBS and the polarizability of anisotropic units (statistical segment length) provides a precise depiction of molecular orientation (Figure 26b).

**Figure 27:**
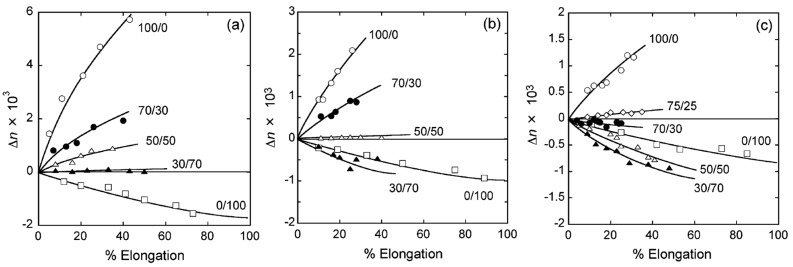
Birefringence of CA/P(VP-*co*-MMA) drawn films prepared at different DS and VP/MMA ratio as a function of elongation: (**a**) DS = 1.80 and VP/MMA = 35:65; (**b**) DS = 2.18 and VP/MMA = 47:53; (**c**) DS = 2.48 and VP/MMA = 50:50. Reprinted with permission from ref. [40]. Copyright 2007 American Chemical Society.

Ohno and Nishio evaluated the molecular orientation and optical anisotropy resulting from the stretching of the already-described miscible blend films of CA and VP-based vinyl polymers [40]. P(VP-*co*-MMA) copolymers showed negative birefringence (Figure 27). Birefringence development in the blends was widely controllable in degree and polarity by altering the DS of CA, the VP/MMA ratio in P(VP-*co*-MMA), and the mixed polymer proportions because the miscible polymer components adopted a cooperative orientation during the uniaxial drawing process (Figure 27). At a specific blend composition (Figure 11), the drawn film behaved like an optically isotropic medium even though it was expected to be mechanically anisotropic after deformation.

**Figure 28:**
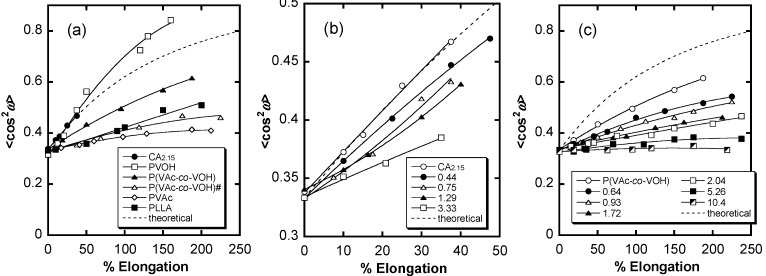
<cos^2^ ω> plots of films comprising CA_2.15_-*g*-PLLA (**a**); PVAVAc-*g*-PLLA (**b**); and their component polymers and related ones (**c**) as a function of elongation. Broken lines represent theoretical curves obeying the Kratky-type affine deformation model. Numerals shown in (b) and (c) represent OH-MS values for graft copolymers. The parameter OH-MS was mainly chosen to characterize the grafting degree, defined as the average number of introduced lactyl units per hydroxyl group in the original CA or PVAVAc used as trunk. PVAVAc# is an additional copolymer displaying a VA/VAc molar ratio of 0.164/0.836. Reproduced with permission from ref. [72]. Copyright 2011 Springer Science + Business Media B.V.

On the other hand, practical properties and functions of cellulosic graft copolymers as thermoplastic solids may change dramatically according to their polymer chain orientation, which results from their manufacturing into geometrically defined products (e.g., films and filaments). Unohara *et al.* examined the stretching-induced molecular orientation and optical anisotropy in CA_2.15_-*g*-PLLA films in connection with their grafted side chain length [72]. To address the rigidity of the trunk polymers, the flexible poly(vinyl acetate-*co*-vinyl alcohol) (PVAVAc; VA/VAc molar ratio = 0.358:0.642) was chosen as an additional trunk polymer instead of the semi-rigid CA_2.15_. The overall orientation was estimated using the statistical second (<cos^2^ ω>) and fourth (<cos^4^ ω>) moments obtained by fluorescence polarization and demonstrated that all stretched films exhibited a positive orientation function (*i.e.*, *f* = ((3<cos^2^ ω> − 1)/2) > 0) that increased with increasing deformation. The degree of molecular orientation was higher in the CA_2.15_ graft series containing a semi-rigid trunk but decreased monotonically with increasing PLLA side chain content in both film types (Figure 28).

**Figure 29:**
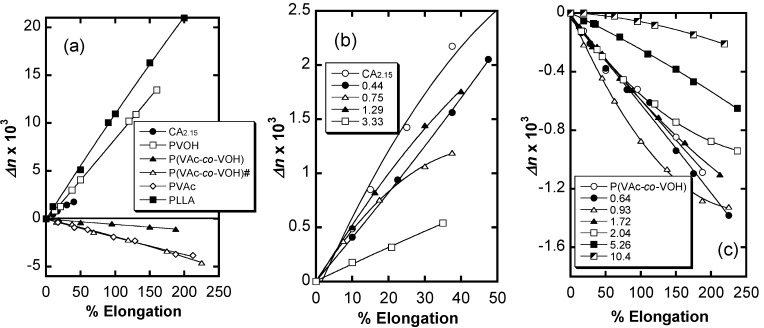
Birefringence plots of films comprising CA_2.15_-*g*-PLLA (**b**), PVAVAc-*g*-PLLA (**c**), and their component polymers and related ones (**a**) as a function of elongation. Numerals in (b) and (c) correspond to OH-MS values for graft copolymers. PVAVAc# is an additional copolymer presenting a VA/VAc molar ratio of 0.164/0.836. Reproduced with permission from ref. [72]. Copyright 2011 Springer Science + Business Media B.V.

**Figure 30:**
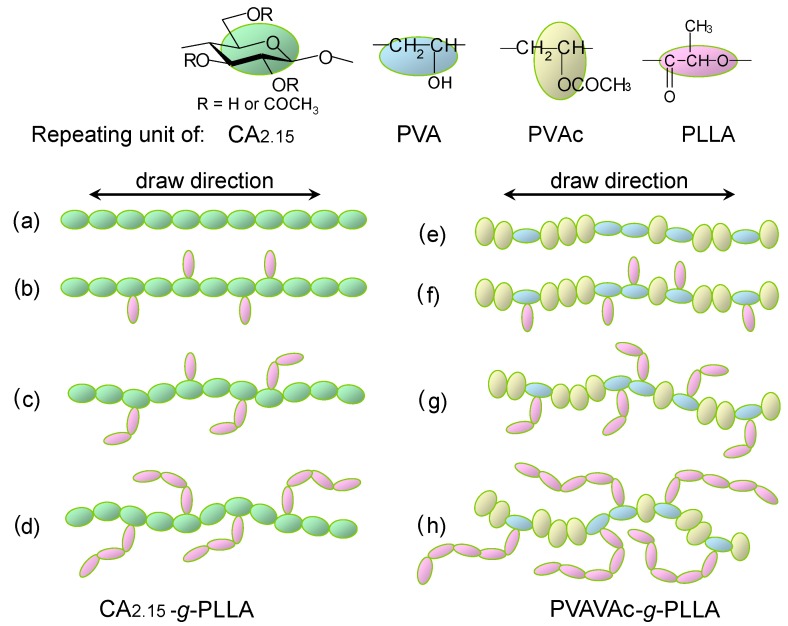
Schematic representation of orientational birefringence in CA_2.15_-*g*-PLLA (**a**–**d**) and PVAVAc-*g*-PLLA (**e**–**h**). Polymeric chains appear as polarizability ellipsoid sequences of the constituent monomeric units. Reproduced with permission from ref. [72]. Copyright 2011 Springer Science + Business Media B.V.

In terms of optical anisotropy (Figure 29), CA_2.15_-*g*-PLLA films constantly exhibited a positive birefringence (*Δn* > 0) upon stretching whereas PVAVAc-*g*-PLLA drawn films displayed a negative value. This contrast in polarity reflects the difference in intrinsic birefringence between the two trunk polymers. Of particular interest was the discovery of a discontinuous change in *Δn* for a given stage of elongation with copolymer composition that is indicated with MS. This may stem from the different localized orientations of the attached PLLA chain segments. Lactyl units positioned close to the graft joint are arranged perpendicular to the trunk chain, which is most closely aligned to the drawing direction, and make a negative contribution to birefringence. On the other hand, lactyl units located far away from the joint are preferentially oriented in the drawing direction and make a positive contribution to the total birefringence. The situation is illustrated schematically in Figure 30.

**Figure 31:**
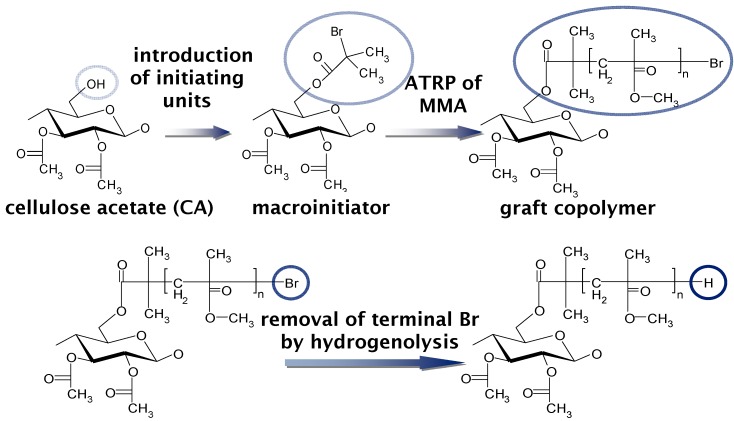
Synthesis of CA-g-PMMA.

**Figure 32:**
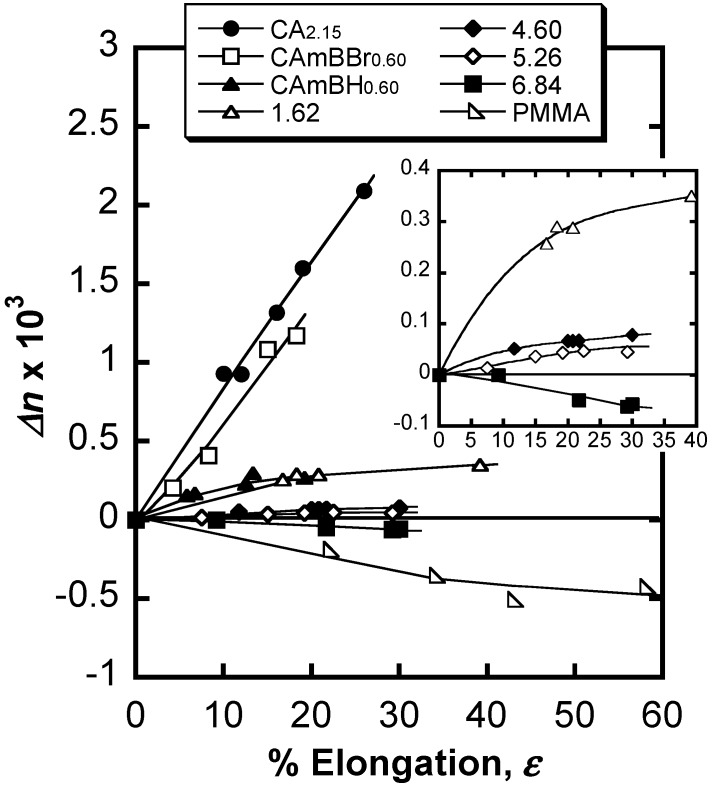
Birefringence plots of films comprising CA-*g*-PMMA copolymers and their component polymers and related ones as a function of elongation. Numerals represent MS values for the CA-*g*-PMMA series. Inset: graft copolymer data on an enlarged scale. Reproduced with permission from ref. [73]. Copyright 2013 American Chemical Society.

**Figure 33:**
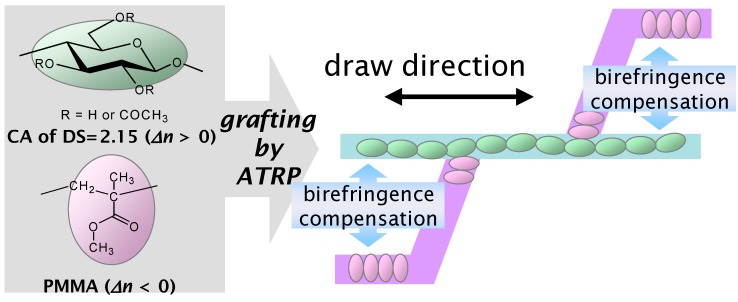
Schematic representation of orientational birefringence in CA-*g*-PMMA. Reproduced with permission from ref. [73]. Copyright 2013 American Chemical Society.

Very recently, Yamanaka *et al.* prepared graft copolymers composed of CA_2.15_ and PMMA at various ratios via atom-transfer radical polymerization (ATRP) using a CuBr/*N*,*N*,*N'*,*N,”N"*-pentamethyldiethylenetriamine catalytic system [73]. PMMA was chosen because of its importance in optical applications. These CA-*g*-PMMA graft copolymers were prepared in three steps (Figure 31), including (a) the ATRP initiator (2-bromoisobutyryl groups) introduction onto CA chains to obtain the macroinitiator CAmBBr, (b) ATRP grafting, and (c) the dehalogenation (hydrogenolysis) of the PMMA side-chain terminal. All reactions were conducted in homogeneous solutions and molecular characterization was performed by NMR and GPC analyses before and after deliberate cleavage of the PMMA grafts. These measurements revealed that the polydispersity index remained below 1.2 even if the graft molecular weight increased up to ~3000. Therefore, the graft chains were clearly produced via a well-controlled ATRP mechanism. The *Δn* value (Figure 32) at any given stage of the copolymer film elongation also decreased rapidly with increasing MS, leading to a transition from the positive *Δn* of pristine CA_2.15_ to a negative value above 65% PMMA (the highest MS in the graft series used). Figure 33 shows a schematic representation of orientational birefringence in CA-*g*-PMMA. These graft copolymers may therefore find use as a highly functional material whose optical anisotropy can be controlled through birefringence compensation between the oriented trunk and graft chains. This material may play a significant role in ensuring molecular orientation growth and facilitate the design of optical retardant films exhibiting zero-birefringence regardless of their elongation.

## 7. Conclusions

This review focuses on the functionalization of cellulose and related polysaccharides as thermoplastic bulk materials. Four major systems, including single-substituent derivatives, derivatives with multiple substituents, polymer blends, and graft copolymers, were addressed. Studies summarized in this article highlighted highly complicated thermal (phase) transition behaviors in these derivatives despite their simple appearance. The accumulation of inclusive data on the relationship between the structure and thermal and mechanical properties is of primary importance for application of structural polysaccharide derivatives as thermoplastic materials. In conjunction with a rising impetus for the development of recyclable and/or renewable materials using biomass, it is a great reassurance that the active utilization of cellulose-based materials is practically conducted by industry, in particular via multiple derivatization. Polysaccharide derivatives utilized as commodity-type products and materials exerting special functions, such as stimulation response, biodegradability, separation, adsorption, and specific mechanical and optical performance, are expected to play crucial roles in numerous fields. Because substituents can be introduced with comparative ease in most cases, the material function added to the polysaccharides is important. This functionalization of polysaccharides will prove fruitful if controlled at the molecular primary and higher-order levels, such as specific phase developments as well as molecular and segmental orientations. Continuing research efforts are expected to expand the industrial use of a wide spectrum of polysaccharide derivative-based materials.

## Abbreviations

ABS: acrylonitrile butadiene styrene
Ac: acetate
Acyl-Ch: acyl chitin
AFM: atomic force microscopy
amide-DS: degree of amide substitution
ATR-FTIR: attenuated total reflection FT-IR
ATRP: atom-transfer radical polymerization
BBS: 4,4'-bis(2-benzoxazolyl)stilbene
BL: (R,S)-β-butyrolactone
Bu: butyrate
CA: cellulose acetate
CB: cellulose butyrate
CD: curdlan
CDA: cellulose diacetate
Cell: cellulose
CE: cellulose ester
ChB: chitin butyrate
CL: ε-caprolactone
CMC: tri-*O*-carboxymethyl cellulose
CP: cellulose propionate
CP/MAS: cross-polarization magic angle-spinning
De: decanoate
DHPC: *O*-(2,3-dihydroxypropyl) cellulose
DMA: dynamic mechanical analysis
DMAc: *N*,*N*-dimethylacetamide
DMSO: dimethyl sulfoxide
*Δn*: birefringence
DPs: average degree of polymerization of side chains
DS: degree of substitution
DSC: differential scanning calorimetry
EC: ethyl cellulose
2-Eh: 2-ethylhexanoyl
ester-DS: degree of ester substitution
FE-SEM: field emission scanning electron microscope
GM: konjac glucomannan
He: hexanoate
IM: immiscible
La: laurate
LC: liquid crystalline
LCD: liquid crystal display
*m*: side chain length, where *m* is defined as the number of the carbon atoms forming the side chain skeleton
M: miscible
M(*t*): magnetization intensity observed as a function of the spin-locking time *t*
MA: mercaptoacetic acid
MMA: methyl methacrylate
bis-MPA: bis(methylol)-2-methylpropanamide
MS: molar substitution
Oc: octanoate
oxyalkanoyl DS: degree of oxyalkanoyl substitution
P(VP-*co*-MMA): poly(*N*-vinyl pyrrolidone-*co*-methyl methacrylate)
PAA: 3-pentadecylphenoxy acetic acid
PCL: poly(ε-caprolactone)
PHA: poly(hydroxy alkanoate)
PLA: poly(lactic acid)
PLLA: poly(l-lactide)
PM: partially miscible
PMMA: poly(methyl methacrylate)
POM: polarized optical microscope
Pr: propionate
PVAVAc: poly(vinyl acetate-*co*-vinyl alcohol)
PVB: poly(vinyl butyral)
PVP: poly(*N*-vinyl pyrrolidone)
*T*_1ρ_^H^: ^1^H spin-lattice relaxation time in the rotating frame
TAC: triacetyl cellulose
*T*_g_: glass transition temperature
total-DS: total degree of subsitution
Va: valerate
VAc: vinyl acetate
VL: δ-valerolactone
VP: *N*-vinyl pyrrolidone
WAXD: wide-angle X-ray diffractometry
XG: xyloglucan
XylC6N_3_: di-*O*-(6-azidohexanoyl)-xylan
